# Performance evaluation and application of a multiplex PCR capillary electrophoresis method for detecting nucleic acids of seven sexually transmitted pathogens

**DOI:** 10.3389/fcimb.2026.1816857

**Published:** 2026-05-18

**Authors:** Yanping Dai, Kang Wang, Yong Wu, Wei Liang

**Affiliations:** 1 Department of Laboratory Medicine, The First Affiliated Hospital of Ningbo University, Ningbo, Zhejiang, China; 2 Reagent Research and Development Center, Ningbo Health Gene Technologies Co., Ltd., Ningbo, Zhejiang, China; 3 Department of Laboratory Medicine, The First Affiliated Hospital of Ningbo University, Ningbo, Zhejiang, China; 4 Zhejiang Engineering Research Center of Innovative Technologies and Diagnostic and Therapeutic Equipment for Urinary System Diseases, Ningbo, Zhejiang, China

**Keywords:** epidemiological research, multiplex PCR capillary electrophoresis, performance evaluation, rapid screening, sexually transmitted diseases

## Abstract

**Objective:**

To establish a rapid multiplex PCR capillary electrophoresis (MPCE) method for the simultaneous detection of seven sexually transmitted infection pathogens, and to evaluate its clinical utility.

**Methods:**

We designed specific primers and optimized multiplex PCR conditions. Capillary electrophoresis enabled fragment analysis. We assessed diagnostic performance, including sensitivity, specificity, anti-interference capacity, and repeatability. Using commercially available reagents as references, we evaluated the agreement of MPCE with 205 clinical samples. Kappa consistency test and McNemar test were used to evaluate the consistency and difference between this method and the reference methods.

**Results:**

The detection system was successfully constructed, with a detection time of three hours. It enabled simultaneous detection of seven common genital tract pathogens, including *Chlamydia trachomatis* (CT), *Ureaplasma urealyticum* (UU), *Mycoplasma genitalium* (MG), *Neisseria gonorrhoeae* (NG), *Mycoplasma hominis* (MH), *Herpes simplex virus type 2* (HSV-2), and *Ureaplasma parvum* (UP). The method demonstrated good specificity and anti-interference capacity, with a limit of detection (LOD) ranging from 325 to 900 copies/mL across all targets. Repeatability results showed minimal fragment length fluctuations (0.13-0.20 nt), and the coefficient of variation (CV) for log peak area ranged from 0.78% to 5.56%. Agreement between the developed method and commercial reference assays was good for all seven pathogens: CT, 94.93% (Kappa = 0.84); MG, 95.12% (Kappa = 0.88); NG, 96.59% (Kappa = 0.91); UP, 95.61% (Kappa = 0.88); MH, 97.07% (Kappa = 0.82); HSV-2, 100% (Kappa = 1.00); and UU, 91.71% (Kappa = 0.69). The positivity rates of MG and NG detected by this method were higher than those by the reference methods (*P* = 0.008 and *P* = 0.011, respectively), and there was no statistical difference for the other pathogens (*P*>0.05).

**Conclusion:**

This study successfully established a MPCE-based assay for the simultaneous detection of seven common genital tract pathogens (CT, UU, MG, NG, MH, HSV-2, and UP) in a single reaction. The method demonstrated high sensitivity, excellent repeatability and strong agreement with commercial reference assays. With its high throughput and rapid turnaround time, this method is well-suited for clinical screening and large-scale epidemiological surveillance of genital tract infections.

## 1 Introduction

Sexually transmitted diseases (STDs) are identifiable disease states that arise from sexually transmitted infections (STIs) (Workowski et al., 2021). They pose a significant epidemiological challenge, profoundly affecting the sexual and reproductive health of both men and women, and may even increase the risk of HIV infection (Ward and Rönn, 2010). The World Health Organization estimates that over one million people aged 15 to 49 worldwide contract curable sexually transmitted infections daily, with most infected individuals showing no symptoms (Wihlfahrt et al., 2023). The growing health burden of sexually transmitted diseases and the escalating costs of prevention and control necessitate rapid and reliable laboratory techniques for identifying causative pathogens (Lee et al., 2007). This reduces the incidence of complications and the risk of disease transmission. However, due to the wide variety of pathogens causing sexually transmitted infections and the common occurrence of mixed infections (Samra et al., 2011; Dupin, 2013; Hangzhen et al., 2025; Xiangcong et al., 2025), researchers have been seeking diagnostic methods capable of simultaneously identifying multiple pathogens within a single clinical sample. With the rapid advancement of biotechnology, polymerase chain reaction (PCR) technology has gained widespread application in pathogen detection due to its speed and high sensitivity. It is particularly suitable for detecting microorganisms that are difficult to culture or cannot be cultured using conventional methods. Multiplex PCR utilizes multiple primer pairs to amplify multiple target sequences in a single reaction, not only enhancing detection efficiency but also significantly reducing costs, thereby enabling simultaneous detection of multiple pathogen targets (Janpoor et al., 2017; Ilami et al., 2013). However, the presence of multiple primer pairs simultaneously increases the risk of primer dimer formation or non-specific amplification, making the design of appropriate, specific primers crucial (Shum and Paul, 2009). Optimizing reaction conditions for multiplex PCR (such as primer concentration and annealing temperature) is critical for experimental success. Inadequate optimization can lead to issues including poor sensitivity, reduced specificity, and uneven amplification of different targets (Elnifro et al., 2000; Markoulatos et al., 2002; Yang and Marchand, 2002; Sint et al., 2012). Additionally, traditional multiplex PCR techniques rely on gel electrophoresis for result analysis. Conventional agarose gel electrophoresis exhibits significant limitations in DNA band resolution and sensitivity, which restricts the application of multiplex PCR in high-throughput detection (Zhong et al., 2020). Capillary electrophoresis is an electrophoretic separation technique that utilizes capillaries as separation channels, achieving separation based on differences in flow rates and distribution behavior among sample components (Heller, 2001). When applied to the analysis of multiplex PCR products, this technique demonstrates performance comparable to high-performance liquid chromatography, offering advantages such as rapidity, automation, high throughput, accurate quantification, and excellent reproducibility (Thalinger et al., 2021; Yi et al., 2025). In response to the current prevalence of STIs and common mixed infections, we established and optimized a multiplex PCR capillary electrophoresis (MPCE) method. By designing specific primers for seven major STI pathogens—*Chlamydia trachomatis* (CT)*, Ureaplasma urealyticum* (UU)*, Mycoplasma genitalium* (MG)*, Neisseria gonorrhoeae* (NG)*, Mycoplasma hominis* (MH)*, Herpes Simplex Virus Type 2* (HSV-2)*, and Ureaplasma parvum* (UP). This method enables multiplex nucleic acid detection through capillary electrophoresis fragment analysis of purified nucleic acids amplified in a single tube. This method can simultaneously detect and distinguish seven common genital tract pathogens while offering favorable cost-effectiveness. Through a series of experiments, we systematically evaluated and validated its performance metrics, including accuracy, sensitivity, specificity, and resistance to interference.

## 2 Materials and methods

### 2.1 Main reagents and materials

PCR Amplification Reagents (UE); Gel Recovery Kit (Magen); SacI-HF Restriction Endonuclease, NedI Restriction Endonuclease (NEB); T4 Ligase (TAKARA); DH5α Competent *E. coli* (Genye Bio); Plasmid Extraction Reagents (TIANGEN); Nucleic Acid Extraction Reagents (Chongqing Zhongyuan Biotechnology Co., Ltd.); Multiplex PCR Amplification Reagents, HiDi Formamide, Size500 Fluorescent Internal Standard, High Molecular Weight Separation Gel (Ningbo Health Gene Technology Co., Ltd.). Pet20(b)+ Plasmid (Department of Laboratory Medicine, The First Affiliated Hospital of Ningbo University). Control Reagents: Nucleic acid detection kit for reproductive tract pathogens (fluorescence PCR method) (Wuhan Easy Diagnosis Biomedicine Co Ltd); Herpes Simplex Virus Type 2 Nucleic Acid Detection Kit (PCR-Fluorescent Probe Method) (Sansure Biotech Inc.).

### 2.2 Primary instruments and equipment

EXM6000 Nucleic Acid Extraction Analyzer (Chongqing Zhongyuan Biotechnology Co., Ltd.); Gentier96R PCR Analyzer (Xi’an Tianlong Biotechnology Co., Ltd.); CE2400 Capillary Electrophoresis Analyzer, Data Acquisition Software (Management Module) Version 1.0.199 (Ningbo Health Gene Technology Co., Ltd.).

### 2.3 Clinical samples

Residual urine samples were collected from patients with suspected reproductive tract infections at our hospital following routine urinalysis. Positive and negative status for each pathogen was confirmed using commercially available reference methods (see section 3.6.5 for details), which served as the basis for subsequent consistency analysis. Inclusion criteria were based on the presence of one or more clinical symptoms suggestive of reproductive tract infection, including urethral discharge, dysuria, frequent urination, genital itching, or lower abdominal pain. They also required a sample volume of at least 500 μL and that the sample was obtained from the patient’s initial examination. Exclusion criteria included insufficient sample volume (<500 μL) and duplicate samples from the same patient. All samples were stored at –20°C for no more than three months and subjected to no more than three freeze-thaw cycles prior to testing. Before testing, samples were restored to room temperature and thoroughly mixed.

## 3 Experimental design

### 3.1 Primer design and selection

Based on the conserved sequences of CT, UU, MH, MG, NG, UP, HSV-2, and the conserved sequence of the human LDHA gene (HIC), Primer-BLAST was used to design specific pathogen primers and internal reference gene primers for sample quality control. Additionally, primers were designed based on Python-randomly generated sequences (IC) to ensure quality control throughout the entire detection process. The PCR product length was designed to range between 100–350 bp to achieve optimal separation and reduce separation time. To ensure sufficient spacing between PCR products from different targets, additional bases were added to the 5’ end of some primers. The fragment size difference between adjacent PCR products was approximately 10–40 nt to prevent peak drag from saturated targets affecting the interpretation of preceding targets.Through rigorous primer sequence alignment, non-specific amplification is avoided, ensuring primers do not cross-amplify between different targets. Primer sequence structures are optimized to minimize the formation of secondary structures such as dimers and hairpin structures, thereby reducing interference between primers. Primers were synthesized by Thermo Fisher Scientific. Each primer pair was modified by labeling the 5’ end of either the forward or reverse primer with fluorescein amide (FAM), a fluorescent dye known for its low cost, stable performance, and broad applicability.

Three sets of detection primers were designed for each target site. Each primer set included primers for seven pathogen target sites and two internal quality control primers (HIC and IC). Nucleic acids extracted from each target site served as templates for amplification, followed by capillary electrophoresis separation. With three replicates per sample, the average amplification efficiency (peak height) of each target site across the three primer sets was compared to select primers with relatively higher amplification efficiency.

### 3.2 Single and multiplex PCR assays

As instructed, the specified volume of ddH_2_O was added to each target primer tube, resulting in a primer solution with an initial concentration of 100 μM. Subsequently, a primer mix with a working concentration of 4 μM was prepared. Using DNA from seven target pathogens as templates, single PCR amplification was conducted for each pathogen with seven pairs of specific primers. A mixture of genomic DNA from the seven target pathogens, along with HIC and IC, served as a template for multiplex PCR amplification with a primer mix.

The PCR reaction system consisted of 20 μL: 5 μL of template DNA, 1 μL of primer (4 μM), 5 μL of 4× PCR Mix, 1 μL of Enzyme Mix, and water added to reach a total volume of 20 μL. Reaction conditions were as follows: incubation at 25°C for 120 seconds, pre-denaturation at 95°C for 120 seconds (1 cycle); denaturation at 94°C for 5 seconds, annealing and extension at 61°C for 45 seconds (31 cycles); extension at 60°C for 10 minutes (1 cycle); and storage at 4°C.

### 3.3 Capillary electrophoresis separation

PCR products were analyzed using capillary electrophoresis according to the following protocol. 1 μL of the PCR product was mixed with 9 μL of Hi-Di formamide containing 2.5% fluorescent internal standard dye. Fragment separation was performed using a CE2400 fully automated capillary electrophoresis analyzer. The analyzer was operated with a polyacrylamide polymer injection voltage of 3.5 kV for 8 seconds and a run voltage of 19.5 kV for 1120 seconds at 60°C. Other electrophoresis parameters are detailed in **Appendix 1, Table 17**. A total of 21 gradients of internal size standards, spanning from 75 nt to 500 nt, were used to calibrate the fragment length of each sample. Electrophoresis patterns and peak profiles were generated through the Data Acquisition Software-Management Module. Fragment length (peak size) was determined by comparing migration times with the internal standard. Pathogen identification was achieved based on the specific peak size of each amplicon, corresponding to the expected fragment length for each target. Peak height, defined as the apex of the peak, provides a rapid estimation of product abundance, although it is influenced by peak width. Peak area, representing the area under the curve, reflects the relative quantity of the amplicon and is appropriate for quantitative analysis, including the calculation of peak ratios and relative target abundance.

### 3.4 Optimization of multiplex PCR systems

The total reaction volume was 20 μL, with a recommended volume of 5 μL for the 4× Reaction Buffer (SEN), including NTPs, magnesium chloride, and buffer components. The volume of the Enzyme Mix (SEN) (containing hot-start DNA polymerase and UDG), and the final concentration of primers for each detection target, were determined through optimization testing to ensure maximum overall sensitivity.

#### 3.4.1 Study on enzyme dosage

In the above reaction system, three different enzyme dosages—0.5 μL, 1 μL, and 1.5 μL—were used. All other components of the system and the reaction conditions remained identical to those outlined in Section 3.2. The impact of varying enzyme quantities on the amplification efficiency of this system was assessed.

#### 3.4.2 Optimization of primer concentration

A pooled primer solution with a concentration of 100 μM was prepared to ensure each target primer was at 4 μM. Subsequently, 1 μL, 1.5 μL, 2 μL, 2.5 μL, and 3 μL of this solution were added to a 20 μL reaction volume, achieving final primer concentrations of 200 nM, 300 nM, 400 nM, 500 nM, and 600 nM, respectively. The optimal enzyme amount identified in Section 3.4.1 was used, while other reaction components and conditions remained consistent with those in Section 3.2. This setup was designed to compare how varying primer concentrations affect the amplification efficiency of each target.

#### 3.4.3 Study on annealing temperature optimization

Using nucleic acids extracted from pathogens at various target sites, IC plasmids, and two clinical samples as templates, the reaction system was established based on the optimized PCR system design results. The reaction conditions were as follows: 120 seconds at 25°C, pre-denaturation at 95°C for 120 seconds, 1 cycle; denaturation at 94°C for 5 seconds, annealing and extension at X°C for 45 seconds, 31 cycles; 60°C for 600 seconds, 1 cycle; hold at 4°C. X was set to 59°C, 60°C, 61°C, 62°C, and 63°C to compare the effects of different annealing temperatures on amplification efficiency for each target.

#### 3.4.4 Annealing time optimization study

Using nucleic acids extracted from pathogens at each target site, the IC plasmid, plasmid mix, and two clinical samples were used as templates. Based on the optimized PCR system design results, the reaction system was established with the following conditions: 25°C for 120 seconds; pre-denaturation at 95°C for 120 seconds (1 cycle); denaturation at 94°C for 5 seconds, annealing-extension at X°C for Y seconds (31 cycles); final extension at 60°C for 600 seconds (1 cycle); and storage at 4°C. X represents the optimal annealing-extension temperature determined in Section 3.4.3. Y was set to 35 seconds, 45 seconds, and 55 seconds to compare the effects of different annealing-extension durations on amplification efficiency for each target during the cycles.

### 3.5 Simulated sample construction

#### 3.5.1 Acquisition of simulated sample fragments

Primers were designed to extend 10-100 base pairs upstream and downstream of the sequence described in Section 3.1. Specific primers for the seven pathogens were designed with Primer-BLAST, and *Sac*I and *Nde*I restriction sites with overhangs were included at both termini. Primer synthesis was carried out by Universal Biosyntheses. PCR amplification was conducted under the conditions described previously. PCR products were resolved on a 1% agarose gel at 110 V and a current of 100 ± 10 mA for 30 minutes. Target bands were excised, DNA was purified using a gel extraction kit, and the concentration of the recovered DNA was measured.

#### 3.5.2 Simulated plasmid recombination

The PCR products and pET-20b(+) vector were simultaneously digested with *Sac*I-HF and *Nde*I at 37°C for 3 hours. The digested products were then separated using 1% agarose gel electrophoresis under the same conditions. Specific bands were excised from the gel, and the DNA concentrations were measured. The amounts of vector and fragment were calculated using the NEBio Calculator, maintaining a molar ratio of vector to fragment of 1:3. T4 ligase-mediated recombination was performed at 4°C for 16 hours. Thirty-three microliters of DH5α *E. coli* were thawed on ice and mixed with 3 μL of the ligation product. Heat shock was applied at 42°C for 90 seconds, immediately followed by a 3-minute ice bath at 0°C. The entire bacterial suspension was spread onto 1% ampicillin-resistant plates and incubated at 37°C for 16–18 hours. Ten single colonies were selected and verified through colony PCR using primers specific to sequences flanking the vector. The PCR products were verified on a 1% agarose gel under the same conditions. Colonies with correctly sized bands were sent to Genecomp for sequencing, resulting in strains containing pathogen-specific sequences. After 16–18 hours of incubation at 37°C, plasmid DNA from the simulated samples was extracted using a commercial plasmid extraction kit (refer to the kit instructions for specific procedures). The mass concentration was measured and converted to copy number concentration (Zeng et al., 2025).

### 3.6 Performance evaluation

#### 3.6.1 Sensitivity

We used the minimum detection limit to evaluate the sensitivity of the experimental method in this study. The method is as follows: The recombinant plasmid DNA solutions of the seven extracted pathogens were first diluted in a 10-fold gradient with enzyme-free water, and each sample was repeated three times. The lowest concentration at which all 3 replicates were detected was defined as the critical detection concentration. Then, the critical detection concentration was diluted in a 2-fold gradient, and each sample was repeated 3 times. Finally, the lowest concentration with a detection rate of 100% was determined as the limit of detection.

#### 3.6.2 Specificity

The specificity of the multiplex assay was evaluated by assessing cross-reactivity both among the seven target pathogens and with non-target microorganisms. First, to exclude cross-reactivity among the seven target pathogens, each primer pair was tested individually against all other six targets (Section 3.2). To comprehensively evaluate the specificity of the detection method established in this study, multiple pathogens with similar clinical manifestations or common in the same biological sample were deliberately screened for cross-reactivity tests. Pathogen samples included *Staphylococcus aureus* (SA), *Staphylococcus epidermidis* (SE)*, Pseudomonas aeruginosa* (PA)*, Klebsiella pneumoniae* (KP)*, Escherichia coli* (EC)*, Candida albicans* (CA) (all at a concentration of 10^6^ CFU/mL), as well as strongly positive samples of *Human papillomavirus type 16* (HPV-16), and *Human papillomavirus type 18* (HPV-18). Notably, the positive samples of HPV-16 and HPV-18 were confirmed to be negative for other targets by commercial kits. Nucleic acid extraction was performed and the detection was repeated three times.

#### 3.6.3 Anti-interference tests

Samples positive for all seven pathogens were respectively spiked with interferents, such as hemoglobin (final concentration: 1.2 mg/mL), mucin (final concentration: 1.2 mg/mL), 1% clotrimazole (final concentration: 0.01%), and 2% miconazole (final concentration: 0.02%), and 10% glycerol (final concentration: 0.1 mg/mL). Nucleic acids were extracted and the detection was repeated three times. The changes in positive results were observed.

#### 3.6.4 Repeatability

Twenty reactions were repeated for each of the two concentrations of the recombinant plasmid simulated samples. The percentage of positive results was calculated, and the changes in the peak size (fragment length) of each target were statistically analyzed to evaluate the repeatability. Although our method is essentially a qualitative judgment (positive/negative), in clinical practice, the change in relative peak area often has a certain correlation with the pathogen load. This correlation is underpinned by the fact that the fragments observed in capillary electrophoresis are the amplified products of the original DNA. Gi Won Shin et al. demonstrated that there is a strong linear correlation between the Lg value of the target peak area in capillary electrophoresis and the Lg value of the original PCR concentration, thus enabling the quantification of the target in the original sample (Shin et al., 2008, 2010). Consequently, we further assessed the method’s repeatability across different concentration levels by verifying the coefficient of variation (CV) of the log-transformed peak areas, a crucial metric for evaluating detection stability.

#### 3.6.5 Clinical consistency

Samples with positive and negative detection results for pathogens such as CT, UU, MH, MG, NG, and UP were compared and verified using the marketed nucleic acid detection kit for reproductive tract pathogens (fluorescent PCR method) produced by Wuhan Mingde Biotech Co., Ltd. The results for HSV-2 were compared and verified using the marketed nucleic acid detection kit for Herpes Simplex Virus Type 2 (PCR fluorescent probe method) produced by Sanxiang Biotech Co., Ltd. Kappa analysis and the Landis & Koch standard were used to evaluate the consistency between the experimental method and the reference method. A Kappa value of 0.61-0.80 indicates significant consistency, and a value of 0.81-1.0 is judged as almost perfect (Landis and Koch, 1977). The McNemar test was used to compare the differences between them.

## 4 Results

### 4.1 Primer design and screening results

Three sets of primers were designed, amplified, and subjected to capillary electrophoresis. The average peak height from three replicate tests for each target was recorded. Results showed that Group B primers (MH, MG, CT) and Group C primers (UU, UP, NG, HSV-2, HIC, IC) exhibited relatively high average peak heights (see **Appendix 1**). Consequently, these primers were selected as the final primers for the multiplex PCR system (**Table 1**).

**Table 1:** List of specific primers for multiplex PCR.

Target	Primer name	Primer (5’-3’)	PCR fragment length (nt)
MG	MG-F	CCTGTTGAGGTTATTAGCTGGATTTGAAG	186
	MG-R	TTTGTTCACATTACCTTCAGACCATAAGC	
NG	NG-F	CCAATCAGGCAGGTGATGCTTT	252
	NG-R	GCATGCTGTCATCAACAGAGAGC	
MH	MH-F	CCATTTAAGCGTGGCGTATGTACTC	173
	MH-R	CCTTACCTCCACGAATTAAAACAACAC	
CT	CT-F	CCTCTAAGCGATCTGGAACATACAGTG	203
	CT-R	AAGTAAGGAGAAGCATATCCAAAATCCCAC	
UU	UU-F	TCTACCTCAACAACCACAAGCTAATC	216
	UU-R	GGTGGTATAGGAGTAGGTTGAGGAT	
UP	UP-F	GCTCTAATCTTTGTTGGTATAATGGTAGGT	234
	UP-R	TAATAATGGTAATATAGCACCAACGTGATAAAC	
HSV-2	HSV-2-F	GTCACAGGCAACCGAATATGTTCTTCG	273
	HSV-2-R	ACAGAGTGACTCAAGTCCTCCAAAAATC	
HIC	HIC-F	TCACTTAGGTGCCTGTGAATA	147
	HIC-R	CACAATGTGGTTAAATACAGACC	
IC	IC-F	CGACCGGTGTGACTACCACTTA	314
	IC-R	CTGGCATCTCTGCACGATCATAC	

MG denotes Mycoplasma genitalium; NG denotes Neisseria gonorrhoeae; MH denotes Mycoplasma hominis; CT denotes Chlamydia trachomatis; UU denotes Ureaplasma urealyticum; UP denotes Ureaplasma parvum; HSV-2 denotes Herpes Simplex Virus Type 2; HIC denotes human-derived internal control (LDHA gene); IC denotes exogenous internal control (randomly generated sequence).

### 4.2 Single and multiplex PCR detection results

**Figure 1:**
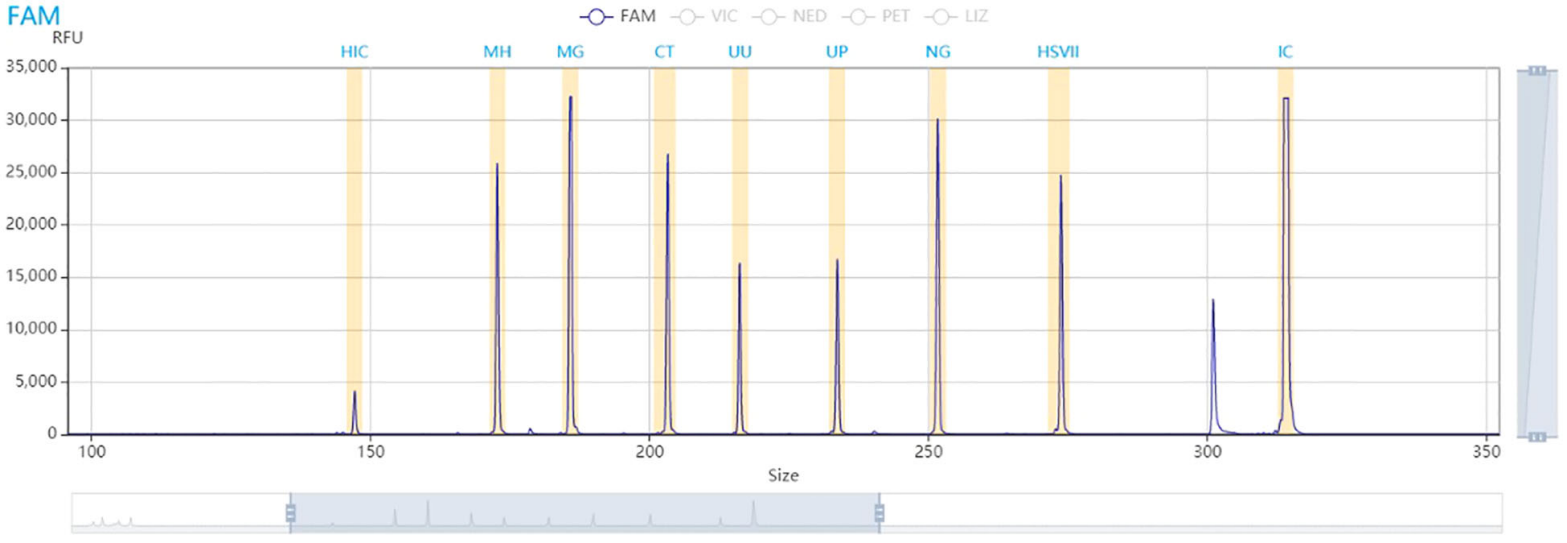
Specific fragment electrophoresis results.

The findings indicate that characteristic amplification peaks appeared at specific positions for CT, UU, MH, MG, NG, UP, and HSV-2 (**Appendix 2, Figures 1–9**). No cross-reactivity was observed among the nine primer pairs; all exhibited distinct, clear peaks at corresponding positions on the electrophoresis patterns. In this study, the minimum interval of the designed band lengths is 13 nt, demonstrating that the resolution of the capillary electrophoresis technology used was adequate to meet the requirements. Experimental results also revealed minor baseline interference peaks and noticeable smear peaks near the internal control (IC). Nonetheless, the designed band differences were sufficient for clear differentiation, did not affect the identification of positive peaks for specific pathogen targets, and had no impact on the results (**Figure 1**).

### 4.3 Optimization results of the multiplex PCR system

#### 4.3.1 Enzyme volume study results

The results indicate that enzyme volumes of 1 µL and 1.5 µL demonstrate higher amplification efficiency, both surpassing that of 0.5 µL. When considering overall material costs, the enzyme volume was set at 1 µL (**Appendix 1, Table 3**).

#### 4.3.2 Primer concentration determination results

All targets exhibited satisfactory detection results at primer concentrations ranging from 300 to 500 nM. A final primer concentration of 400 nM was selected as the optimal one (**Appendix 1, Table 4**).

#### 4.3.3 Annealing temperature optimization results

The PCR annealing temperatures within the range of 59–63°C resulted in relatively stable overall amplification efficiency. Given the need for annealing temperature tolerance, the annealing temperature was set at 61°C (**Appendix 1, Table 5**).

#### 4.3.4 Annealing time optimization results

The amplification efficiency was low when the extension time in the cycle was 35 seconds. There was minimal difference in amplification efficiency between extension times of 45 seconds and 55 seconds. Considering the overall impact on program duration, an annealing time of 45 seconds was chosen for the PCR reaction program (**Appendix 1, Table 6**).

### 4.4 Construction of simulated samples

Following the transformation of recombinant plasmids for seven pathogens, monoclonal colonies were selected from PCR-amplified electrophoresis bands (refer to the electrophoresis diagram) for sequencing. The sequence results indicated consistent alignment (**Appendix 2, Figures 10–23**), confirming correct plasmid recombination. The cultures were then shaken, collected, and the relevant plasmids were extracted. The plasmid concentration was measured using a micro UV spectrophotometer and converted to copy number concentration.

### 4.5 Performance specifications

#### 4.5.1 Sensitivity results

Based on gradient dilution testing results, the minimum detection limits are as follows: MH: 325 copies/mL; MG: 675 copies/mL; CT: 750 copies/mL; UU: 325 copies/mL; UP: 900 copies/mL; NG: 550 copies/mL; HSV-2: 400 copies/mL.

#### 4.5.2 Specificity results

Cross-reactivity among the seven target pathogens was previously assessed during primer validation (Section 3.2), and no nonspecific amplification was observed (**Appendix 2, Figures 1–9**). For non-target testing, the results indicated no cross-reactivity when pathogens such as SA, SE, PA, KP, EC, CA, HPV-16, and HPV-18 were introduced to negative samples, while maintaining well-controlled background noise (**Appendix 1, Table 8**).

#### 4.5.3 Interference testing

Samples positive for all seven pathogens were spiked with interferents—including hemoglobin (1.2 mg/mL), mucin (1.2 mg/mL), clotrimazole (0.01%), miconazole (0.02%), and glycerol (0.1%)—and tested in triplicate. All samples consistently yielded positive results, indicating that the assay exhibits strong resistance to these interferents. (**Appendix 1, Table 9**).

#### 4.5.4 Repeatability results

Twenty replicate reactions were conducted at two concentrations using recombinant plasmid simulated samples. All samples showed a 100% positive rate. The maximum peak size (fragment length) fluctuation ranges for each pathogen are as follows: CT, 0.2 nt; HSV-2, 0.19 nt; MG, 0.15 nt; MH, 0.13 nt; NG, 0.15 nt; UP, 0.14 nt; and UU, 0.14 nt. Statistical analysis of the mean values and coefficients of variation (CVs) for key parameters (peak size, peak height, peak area) of positive peaks revealed that CVs for peak size at both high and low concentrations ranging from 0.01% to 0.02% across all pathogen targets, indicating excellent repeatability. For all targets, the CV values for both log peak height and log peak area were higher at low concentrations compared to high concentrations, which is consistent with analytical chemistry principles. At low concentrations, CV values for log peak area ranged from 1.78% (MG) to 5.56% (UU), while at high concentrations, they ranged from 0.78% (MG) to 2.42% (UU) (**Table 2**). These results demonstrate that the method exhibits good repeatability across the tested concentration ranges. Overall, all CV values remained within an acceptable range, indicating that the detection system maintains good stability and repeatability across different concentration levels. Nonetheless, we emphasize that this method still adheres to the “positive/negative” interpretation criteria in clinical applications.

**Table 2:** Repeatability results.

Target	Peak size (nt)		Log peak height (CV)		Log peak area (CV)	
Target	H	L	H	L	H	L
CT	203.12	203.14	1.61%	3.06%	1.72%	2.77%
HSV-2	273.88	273.90	0.15%	2.64%	1.21%	2.33%
MG	185.92	185.95	0.04%	1.79%	0.78%	1.78%
MH	172.86	172.86	1.83%	2.79%	1.73%	2.51%
NG	252.04	252.03	2.59%	3.06%	2.39%	2.67%
UP	233.80	233.78	2.28%	3.96%	1.91%	3.50%
UU	216.33	216.32	2.74%	6.64%	2.42%	5.56%

Peak size values are presented as means. H, high concentration level; L, low concentration level.

#### 4.5.5 Clinical consistency results

We used 205 clinical samples to validate the consistency of the method. The positive and negative distributions are presented in **Table 3**. Agreement between the developed method and commercial reference assays was good for all seven pathogens: CT, 94.93% (Kappa = 0.84); MG, 95.12% (Kappa = 0.88); NG, 96.59% (Kappa = 0.91); UP, 95.61% (Kappa = 0.88); MH, 97.07% (Kappa = 0.82); HSV-2, 100% (Kappa = 1.00); and UU, 91.71% (Kappa = 0.69). According to the Landis and Koch criteria, the agreement was classified as almost perfect for CT, MG, NG, UP, and MH (Kappa > 0.80), nearly perfect for HSV-2 (Kappa = 1.00), and substantial for UU (Kappa = 0.69). McNemar’s test indicated no significant differences between the new method and the commercial reference reagents for CT, UU, MH, UP, and HSV-2 (*P* > 0.05). The new method, however, showed higher positive rates for NG (*P* = 0.008) and MG (*P* = 0.011) (**Table 4**).

**Table 3:** Distribution of pathogens.

Target	Positive	Negative	Total
CT	50	155	205
MG	59	146	205
MH	20	185	205
NG	56	149	205
UP	52	153	205
UU	35	170	205
HSV-2	3	202	205

**Table 4:** Evaluation of diagnostic agreement.

Target	Agreement rate	Kappa	95%CI	Kappa P^i^	Consistency strength^ii^	Inconsistent pair (b/c)	McNemar P
CT	94.63%	0.86	(0.78, 0.94)	< 0.001	almost perfect	11	0.132
NG	96.59%	0.91	(0.85, 0.98)	< 0.001	almost perfect	7	**0.008** ^*^
MG	95.12%	0.88	(0.80, 0.95)	< 0.001	almost perfect	10	**0.011** ^*^
UU	91.71%	0.69	(0.55, 0.83)	< 0.001	substantial	17	0.225
MH	97.07%	0.82	(0.68, 0.96)	< 0.001	almost perfect	6	0.103
UP	95.61%	0.88	(0.81, 0.96)	< 0.001	almost perfect	9	0.739
HSV-2	100.00%	1.00	(1.00, 1.00)	< 0.001	perfect	0	1.000

^i^ The Kappa *P* value (two-sided) tests whether P<0.05, indicating statistically significant consistency; ^ii^ The consistency strength classification follows the Landis & Koch standard. *Bold indicates *P* < 0.05.

## 5 Discussion

Pathogen detection in clinical settings relies on serology, microscopy, culture, and molecular methods. However, serological markers often lack precision due to limitations like the window period, leading to empirical antibiotic therapy and antimicrobial resistance. Although culture is the gold standard for pathogen detection, it has drawbacks such as low sensitivity, contamination risks, long turnaround times, and challenges in isolating certain organisms like NG (Meyer and Buder, 2020). Factors like antibiotic presence can lead to false negatives, and testing for HSV requires specialized facilities and personnel. Fluorescent probe-based multiplex PCR addresses the specificity and sensitivity challenges of previous methods, allowing for quick, simultaneous pathogen screening. However, each target requires a uniquely labeled fluorescent probe, and the costs of probe synthesis and optimization rise significantly with increased multiplexing. Additionally, using multiple fluorophores in a single reaction can increase fluorescence background and cause spectral crosstalk. Furthermore, PCR instruments generally have a limited number of fluorescence detection channels, usually accommodating only 4–6 targets, with one channel often set aside for internal quality control (Yang and Rothman, 2004). Recent advancements in nucleic acid mass spectrometry and high-throughput sequencing have significantly enhanced ultra-broad-spectrum pathogen detection and typing. These technologies also facilitate the simultaneous identification of multiple drug-resistance genes, providing valuable therapeutic guidance in clinical settings (Charretier and Schrenzel, 2016; Hilt and Ferrieri, 2022; Yang et al., 2024). However, the complexity of the workflows, the need for sophisticated instrumentation, and the relatively high costs restrict their widespread use.

This method is highly time-efficient, with a turnaround time as short as three hours — substantially outperforming culture-based methods (3–5 days) — and reduces testing costs compared to single-pathogen detection, supporting its suitability for widespread implementation. Traditional culture methods cannot isolate all seven pathogens in a single assay, and their detection rates are often compromised by antibiotic interference. In this study, MPCE demonstrated the ability to detect multiple co-infections. For pathogens suppressed but not completely eradicated by antibiotics, culture may yield false-negative results; however, MPCE can still detect their nucleic acids (Wang et al., 2024). Thus, MPCE is particularly valuable for culture-negative specimens and samples collected post-antibiotic treatment, significantly improving detection rates.

Our research indicates that this method exhibits high sensitivity and specificity and detected significantly higher positivity rates for MG and NG compared to the reference methods (*P* = 0.008 and *P* = 0.011, respectively). We reviewed the discrepant positive cases and correlated them with clinical findings and other test results. All patients had symptoms of urinary tract inflammation or infection, and STI pathogens RNA was detected by the Rendu isothermal amplification method on the day the samples were collected. Nine samples were MPCE-positive for MG but negative by the reference method. Of these, four were MG RNA-positive, consistent with the MPCE results, and exhibited relatively high peak areas (1511–22207 RFU; mean 5120 RFU). The remaining five were MG RNA-negative and showed lower MPCE peak areas (315–2792 RFU; mean 442 RFU). Seven samples were MPCE-positive for NG DNA yet negative by the reference method. One of these was NG RNA-positive (peak area 4272 RFU), and six were NG RNA-negative with relatively low peak areas (542–1221 RFU; mean 747 RFU). Although the RNA and DNA assays target different molecules, reference-negative cases with high MPCE peak areas also produced positive RNA results, further suggesting that MPCE may be more sensitive than the reference method. This difference likely reflects the superior analytical sensitivity of capillary electrophoresis, which enables detection of low-abundance amplification products that may be missed by conventional gel-based or less optimized commercial assays. Additionally, the multiplex system was carefully optimized to balance amplification efficiency across all seven targets, potentially improving detection of MG and NG. Detection of nucleic acids at very low concentrations may also result from residual DNA of non-viable pathogens or colonizing bacteria, which may have limited clinical significance. It remains unclear whether patients with such low-level positivity require treatment or whether the condition would resolve spontaneously. Therefore, the clinical significance of detecting very low bacterial loads warrants further investigation.

While this detection method performs well, it has inherent limitations. First, its detection capability is entirely dependent on the coverage of the pre-designed primer panel, which may fail to identify rare mutant strains. In the multiplex PCR reaction stage, competition among multiple primers for limited resources can introduce amplification bias, favoring targets with higher primer binding efficiency, optimal sequence complementarity, and greater abundance. This bias increases the risk of false-negative results for low-abundance or mutant strains (Markoulatos et al., 2002). Second, multiplex PCR capillary electrophoresis detection methods are highly sensitive, which may detect clinically insignificant levels, making it difficult to distinguish between colonization and infection states (Tröger et al., 2016). Moreover, while these methods are rapid and highly sensitive, they cannot replace culture-based approaches for antimicrobial susceptibility testing (Buchan and Ledeboer, 2014). Another technical concern is the risk of contamination: multiple PCR amplifications can increase template quantities by millions of fold, and the sample loading procedure prior to capillary electrophoresis involves opening the reaction vessel, which may generate aerosols containing amplified products, posing a contamination risk that could lead to false-positive results. Beyond these inherent technical constraints, several limitations specific to this study should also be acknowledged. Primarily, this is a single-center study with a relatively modest sample size, which may limit the generalizability of our findings. Furthermore, this study focused solely on diagnostic consistency with reference methods and did not incorporate clinical outcome data; consequently, the relationship between MPCE results and patient treatment responses remains to be established.

In summary, MPCE is a powerful and effective method particularly suited for rapid, large-scale screening of predefined targets. Its benefits include rapid processing, precise resolution, and automated operation, establishing it as a widely used and well-established in molecular diagnostics. However, its application is constrained by inherent limitations such as complex primer design, finite multiplexing capacity, and the inability to perform antimicrobial susceptibility testing. This approach is recommended as a complementary tool for sexually transmitted infection screening, working in tandem with existing traditional methods such as culture to provide more comprehensive pathogen diagnostic information, thereby facilitating targeted antimicrobial therapy and offering data support for epidemiological research.

Looking forward, the strong linear relationship between the log peak area in PCR capillary electrophoresis and the log initial nucleic acid concentration (Shin et al., 2008, 2010) holds promise for enabling precise quantification of pathogen nucleic acids. Such quantitative information could assist in clinical assessment of active infection status, evaluation of antimicrobial treatment efficacy, and prediction of disease prognosis. Furthermore, addressing the increasingly serious challenge of antimicrobial resistance in sexually transmitted pathogens (Unemo and Shafer, 2014; He et al., 2023; Ren et al., 2025) represents a significant application potential by expanding detection targets—such as resistance genes—based on the pathogen spectrum identified in this study.

## Data Availability

The datasets presented in this study can be found in online repositories. The names of the repository/repositories and accession number(s) can be found in the article/**Supplementary Material**.
