# Supplementary material for: Performance evaluation and application of a multiplex PCR capillary electrophoresis method for detecting nucleic acids of seven sexually transmitted pathogens
Source: Front Cell Infect Microbiol. 2026 May 18;16:1816857. doi: 10.3389/fcimb.2026.1816857 (PMC13223153; doi:10.3389/fcimb.2026.1816857)
Supplement: Supplementary file 1 [file Table1.pdf]

Table 1. Amplification efficiency results of three primer sets

Target	Repeat No.	Peak height (RFU) of different primers		
		Set 1	Set 2	Set 3
MH	1	26148	19939	20444
	2	12150	19362	10097
	3	16281	16589	11412
	Mean ± SD	18193 ± 7192	18630 ± 1791	13984 ± 5633
MG	1	22191	32816	12203
	2	32684	32698	14353
	3	16321	32649	13651
	Mean ± SD	23732 ± 8290	32721 ± 86	13402 ± 1096
CT	1	9924	16344	4621
	2	19387	11745	19925
	3	3954	17741	4145
	Mean ± SD	11088 ± 7782	15277 ± 3137	9564 ± 8976
UU	1	21656	22038	25825
	2	21551	14591	20544
	3	30366	18587	32308
	Mean ± SD	24524 ± 5059	18405 ± 3727	26226 ± 5892
UP	1	16664	27228	18624
	2	25386	27427	26000
	3	19491	11320	32609
	Mean ± SD	20514 ± 4450	21992 ± 9242	25744 ± 6996
NG	1	16810	20757	23393
	2	26637	26873	27613
	3	10492	18826	19302
	Mean ± SD	17980 ± 8136	22152 ± 4201	23436 ± 4156
HSV 2	1	25307	28741	32594
	2	26378	18980	14669
	3	16538	12639	29023
	Mean ± SD	22741 ± 5399	20120 ± 8111	25429 ± 9488
IC	1	32566	32582	32584
	2	32627	32732	32686
	3	32626	32629	32636
	Mean ± SD	32606 ± 35	32648 ± 77	32635 ± 51
HIC	1	19164	20444	29300
	2	18541	24362	28948
	3	16553	13810	28487
	Mean ± SD	18086 ± 1364	19539 ± 5334	28912 ± 408

Table2 Simulated sample specific primers

Target	Primer name	Sequence(5'to3')	Length of PCR product（bp）
NG	NG-F-Ndel	CTCACATATGATTCTCAAAGCAACAATCGT	329
	NG-R-SacI	GTCACTGAGCTCTCCCATTTTTCCGCATCTT	
CT	CT-F-Ndel	GTCACATATGGAGAAACATTTCCTCTAAGCG	278
	CT-R-SacI	CACAGAGCTCAAACCTGGCTATATTCTAC	
MG	MG-F-Ndel	GTCACATATGCCTGTTGAGGTTATTAGCTG	204
	MG-R-SacI	GTCTGAGCTCTGTTCACATTACCTTCAGACC	
HSV-2	HSV-2-F-Ndel	CTGACTCATATGTGGGGGGCGTGGG	348
	HSV-2-R-SacI	GACTCAGAGCTCGCGCGTCGTTGTTTC	
UU	UU-F-Ndel	CTGACACATATGAAAAGTAATTATTAAAGACAAT	294
	UU-R-SacI	CAGTCAGAGCTCGGTTCTGGTTCAAC	
UP	UP-F-Ndel	CTGACACATATGGAATAAAAATTAATTTTCAAATT	310
	UP-R-SacI	CTGACTGAGCTCTTATAAATGAAATATGGACC	
MH	MH-F-Ndel	CTGACTCATATGTGCTATGCCCGCACC	258
	MH-R-SacI	GACTCAGAGCTCGTTCCACGAACGATTGTG	

Table 3 Optimization results of enzyme concentration

Sample	Target	Repeat No.	Peak height (RFU) at indicated enzyme dose		
			0.5μL	1μL	1.5μL
Single pathogen nucleic acid	MH	1	22707	31463	15463
		2	21041	17774	27072
		3	22445	18562	18720
		Mean ± SD	22064 ± 896	22600 ± 7686	20418 ± 5988
	MG	1	31385	30305	32217
		2	27226	32168	32091
		3	32101	25910	32121
		Mean ± SD	30237 ± 2632	29461 ± 3213	32143 ± 66
	CT	1	24873	18316	19725
		2	10481	19291	23321
		3	23891	31540	23136
		Mean ± SD	19748 ± 8041	23049 ± 7370	22061 ± 2025
	UU	1	14301	15991	15989
		2	13611	11901	26919
		3	11550	22223	27237
		Mean ± SD	13154 ± 1431	16705 ± 5198	23382 ± 6404
	UP	1	28877	21213	23118
		2	19883	23015	23552
		3	12545	23473	20480
		Mean ± SD	20435 ± 8180	22567 ± 1195	22383 ± 1663
	NG	1	14967	21024	25231
		2	15331	18614	20137
		3	32213	19602	14843
		Mean ± SD	20837 ± 9854	19747 ± 1211	20070 ± 5194
	HSV-2	1	14714	18294	32206
		2	30317	15126	27293
		3	19392	13629	25733
		Mean ± SD	21474 ± 8007	15683 ± 2382	28411 ± 3378
Plasmid Mix	MH	1	6128	27356	15884
		2	N/A	8130	20618

Sample	Targ et	Repeat No.	Peak height (RFU) at indicated enzyme dose		
			0.5μL	1μL	1.5μL
		3	1670	25826	28356
		Mean ± SD	3899 ± 3152	20437 ± 10686	21619 ± 6296
	MG	1	6324	29007	17533
		2	600	12473	19334
		3	1643	32247	30650
		Mean ± SD	2856 ± 3049	24576 ± 10606	22506 ± 7110
	CT	1	7271	16068	11611
		2	N/A	9967	13893
		3	1505	26649	29622
		Mean ± SD	4388 ± 4077	17561 ± 8441	18375 ± 9807
	UU	1	2424	18693	7415
		2	N/A	5172	12132
		3	1103	16317	15904
		Mean ± SD	1764 ± 934	13394 ± 7219	11817 ± 4253
	UP	1	3471	16159	16196
		2	N/A	10854	10595
		3	1084	16656	24900
		Mean ± SD	2278 ± 1688	14556 ± 3216	17230 ± 7208
	NG	1	4342	19861	17288
		2	N/A	17951	20516
		3	1281	30094	32044
		Mean ± SD	2812 ± 2164	22635 ± 6530	23283 ± 7757
	HSV II	1	3650	12432	15206
		2	N/A	12429	17127
		3	666	24717	17825
		Mean ± SD	2158 ± 2110	16526 ± 7094	16719 ± 1356
	IC	1	26768	31967	32084
		2	1704	32232	32240
		3	6958	32113	31873
		Mean ± SD	11810 ± 13218	32104 ± 133	32066 ± 184

Table 4. Optimization results of primer concentrations

Sample Type	Target	Repeat No.	Peak height at indicated primer concentration (nM)				
			200	300	400	500	600
Single pathogen nucleic acid	MH	1	8647	13880	6935	10410	5238
		2	4536	10370	7959	9159	10528
		3	6471	8821	6622	9772	9386
		Mean ± SD	6551 ± 2057	11024 ± 2592	7172 ± 699	9780 ± 626	8384 ± 2784
	MG	1	16687	14777	16739	19535	18396
		2	21616	16790	12360	17044	16051
		3	13313	22190	17587	11083	9705
		Mean ± SD	17205 ± 4176	17919 ± 3833	15562 ± 2805	15887 ± 4343	14717 ± 4496
	CT	1	10458	12888	14056	8220	7815
		2	8841	5568	9714	12581	4626
		3	8501	11133	11317	11686	10653
		Mean ± SD	9267 ± 1046	9863 ± 3822	11696 ± 2196	10829 ± 2303	7698 ± 3015
	UU	1	2408	6093	9563	11128	11493
		2	4361	7079	6824	9462	9246
		3	2495	6299	9635	10842	5709
		Mean ± SD	3088 ± 1103	6490 ± 520	8674 ± 1603	10477 ± 891	8816 ± 2916
	UP	1	4368	6842	14448	11797	12841
		2	7502	10734	11145	10186	9257
		3	4698	10556	6812	11005	11899
		Mean ± SD	5523 ± 1722	9377 ± 2197	10802 ± 3830	10996 ± 806	11332 ± 1858
	NG	1	3261	12099	6886	6005	11230
		2	5653	8615	15129	9126	5622
		3	11241	13260	10331	11625	7416
		Mean ± SD	6718 ± 4095	11325 ± 2417	10782 ± 4140	8919 ± 2816	8089 ± 2864
	HSV-2	1	3920	6357	3215	7621	5049
		2	1298	4264	9008	5368	4817
		3	3169	4504	9275	12175	8177
		Mean ± SD	2796 ± 1350	5042 ± 1145	7166 ± 3424	8388 ± 3468	6014 ± 1877
Plasmid Mix-1	MH	1	9455	11205	16497	13217	4126
		2	9864	6321	14916	9038	6891

		3	8612	12539	16606	11756	8947
		Mean ± SD	9310 ± 638	10022 ± 3274	16006 ± 946	11337 ± 2121	6655 ± 2419
	MG	1	10372	13238	18573	12686	5331
		2	12829	12343	12086	6215	5650
		3	6708	12582	21296	16584	15320
		Mean ± SD	9970 ± 3080	12721 ± 463	17318 ± 4731	11828 ± 5237	8767 ± 5677
	CT	1	8715	11452	8773	8913	8001
		2	9173	9306	8575	8676	4705
		3	6524	7738	13507	11798	10017
		Mean ± SD	8137 ± 1416	9499 ± 1864	10285 ± 2792	9796 ± 1738	7574 ± 2682
	UU	1	973	6996	6630	3774	3279
		2	2749	2968	3917	6836	N/A
		3	1427	3760	6611	7693	3789
		Mean ± SD	1716 ± 923	4575 ± 2134	5719 ± 1561	6101 ± 2060	3534 ± 361
	UP	1	5262	9457	16192	4393	6210
		2	8056	6121	10816	10012	1895
		3	5222	11044	10071	13450	10164
		Mean ± SD	6180 ± 1625	8874 ± 2513	12360 ± 3340	9285 ± 4572	6090 ± 4136
	NG	1	11315	14582	13066	8735	6159
		2	10637	9801	14260	8156	10787
		3	7570	13490	8901	8376	13564
		Mean ± SD	9841 ± 1995	12624 ± 2505	12076 ± 2813	8422 ± 292	10170 ± 3741
	HSV-2	1	3389	6956	5783	6810	3059
		2	4458	5933	4988	7053	1443
		3	2683	3238	7950	4096	5543
		Mean ± SD	3510 ± 894	5376 ± 1921	6240 ± 1533	5986 ± 1642	3348 ± 2065
	IC	1	32199	32159	32125	32121	30463
		2	32155	32194	32158	32263	32161
		3	32271	32097	32146	32196	32142
		Mean ± SD	32208 ± 59	32150 ± 49	32143 ± 17	32193 ± 71	31589 ± 975
Plasmid Mix-2	MH	1	2197	4816	5569	6535	5625
		2	930	7150	2217	14941	1496
		3	6439	5840	5303	6519	4795

	Mean ± SD	3189 ± 2885	5935 ± 1170	4363 ± 1863	9332 ± 4858	3972 ± 2184
MG	1	7106	11491	13013	5291	10292
	2	4717	17424	10484	18639	3827
	3	13860	17369	12233	15124	6034
	Mean ± SD	8561 ± 4742	15428 ± 3410	11910 ± 1295	13018 ± 6919	6718 ± 3286
CT	1	3589	5998	9165	5342	7980
	2	7016	3317	5658	9041	2683
	3	4497	6064	5793	8022	5152
	Mean ± SD	5034 ± 1775	5126 ± 1567	6872 ± 1987	7468 ± 1911	5272 ± 2651
UU	1	1580	4027	6721	2986	5717
	2	1482	6639	2173	6311	1005
	3	1539	1826	3432	3653	7530
	Mean ± SD	1534 ± 49	4164 ± 2409	4109 ± 2348	4317 ± 1759	4751 ± 3368
UP	1	3696	6451	8809	3290	2888
	2	1879	5087	5262	5787	832
	3	2367	4305	10394	7253	7742
	Mean ± SD	2647 ± 940	5281 ± 1086	8155 ± 2628	5443 ± 2004	3821 ± 3548
NG	1	7197	6324	5504	7151	5806
	2	5090	9271	3479	10424	1990
	3	4781	3485	4530	7207	2917
	Mean ± SD	5689 ± 1315	6360 ± 2893	4504 ± 1013	8261 ± 1874	3571 ± 1990
HSV-2	1	2548	9090	10723	2283	7720
	2	2295	5698	6174	15763	2459
	3	6370	7056	6279	12449	7324
	Mean ± SD	3738 ± 2283	7281 ± 1707	7725 ± 2597	10165 ± 7024	5834 ± 2930
IC	1	1069	9764	6753	3583	5215
	2	4828	6002	3006	10413	1093
	3	5378	5143	4332	8303	7122
	Mean ± SD	3758 ± 2345	6970 ± 2458	4697 ± 1900	7433 ± 3497	4477 ± 3082

Table 5 Optimization results of annealing temperature

Sample	Target	Repeat No.	Peak height(RFU)at indicated annealing temperature				
			59℃	60℃	61℃	62℃	63℃
Single pathogen nucleic acid	MH	1	8956	6019	6447	10445	4664
		2	9045	5594	7892	6392	4042
		3	4462	4912	7813	7585	7090
		Mean ± SD	7488 ± 2621	5508 ± 558	7384 ± 812	8141 ± 2083	5265 ± 1611
	MG	1	10335	3449	2986	3659	3116
		2	2479	2960	4629	4160	4010
		3	6450	5546	2354	6820	4130
		Mean ± SD	6421 ± 3928	3985 ± 1374	3323 ± 1174	4880 ± 1699	3752 ± 554
	CT	1	5418	5050	7123	3239	5528
		2	5148	8295	3933	14501	4047
		3	5094	5484	6722	8596	6972
		Mean ± SD	5220 ± 174	6276 ± 1762	5926 ± 1738	8779 ± 5633	5516 ± 1463
	UU	1	14274	7838	9687	8125	4355
		2	10468	9148	5847	4629	3503
		3	15776	8471	8218	6640	3994
		Mean ± SD	13506 ± 2736	8486 ± 655	7917 ± 1938	6465 ± 1755	3951 ± 428
	UP	1	9717	9404	7687	10301	8295
		2	8011	11395	8193	7728	7456
		3	13645	5571	10186	13767	7857
		Mean ± SD	10458 ± 2889	8790 ± 2960	8689 ± 1321	10599 ± 3030	7869 ± 420
	NG	1	4379	1790	3582	8258	4299
		2	8069	1451	2814	4836	3820
		3	4058	3282	5545	2307	4656
		Mean ± SD	5502 ± 2229	2174 ± 974	3980 ± 1408	5134 ± 2987	4258 ± 419
	HSV-2	1	2108	3198	1960	4055	3696
		2	3473	1260	3423	3299	1366
		3	1699	2992	3165	2388	2366
		Mean ± SD	2427 ± 929	2483 ± 1064	2849 ± 781	3247 ± 835	2476 ± 1169
Plasmid Mix	MH	1	609	1276	2006	2261	1680
		2	3230	6233	3176	1958	2077
		3	3889	2244	3901	2593	1569

	Mean ± SD	2576 ± 1735	3251 ± 2627	3028 ± 956	2271 ± 318	1775 ± 267
MG	1	8367	6878	6579	4323	3273
	2	4769	7109	8032	6041	4175
	3	4746	7357	3601	3185	4222
	Mean ± SD	5961 ± 2084	7115 ± 240	6071 ± 2259	4516 ± 1438	3890 ± 535
CT	1	5213	2197	1136	2351	606
	2	1143	7124	5383	1897	860
	3	2703	1301	2961	790	880
	Mean ± SD	3020 ± 2053	3541 ± 3135	3160 ± 2130	1679 ± 803	782 ± 153
UU	1	2572	2096	2808	2903	1188
	2	2034	3264	3118	1981	921
	3	2675	3373	3905	1724	589
	Mean ± SD	2427 ± 344	2911 ± 708	3277 ± 566	2203 ± 620	899 ± 300
UP	1	1889	1550	1600	2480	926
	2	3092	684	2922	1260	1010
	3	1279	1878	1066	2747	2069
	Mean ± SD	2087 ± 923	1371 ± 617	1863 ± 955	2162 ± 793	1335 ± 637
NG	1	2349	2407	2539	1021	1633
	2	3556	1621	6178	1308	909
	3	3365	2807	2361	1155	3063
	Mean ± SD	3090 ± 649	2278 ± 603	3693 ± 2154	1161 ± 144	1868 ± 1096
HSV-2	1	7016	6236	2468	3299	2750
	2	5226	4024	7221	5234	2603
	3	5386	2895	4172	2447	5227
	Mean ± SD	5876 ± 991	4385 ± 1700	4620 ± 2408	3660 ± 1428	3527 ± 1474
IC	1	2913	2932	2137	1364	881
	2	2457	2135	1694	1234	1747
	3	3470	2375	4729	3243	1293
	Mean ± SD	2947 ± 507	2481 ± 409	2853 ± 1639	1947 ± 1124	1307 ± 433

Table 6. Optimization results of annealing time

Sample type	Target	Repeat No.	Peak height(RFU)at indicated annealing time		
			35s	45s	55s
Single pathogen nucleic acid	MH	1	7625	5971	4056
		2	5113	7074	9822
		3	6811	10370	6626
		Mean ± SD	6516 ± 1282	7805 ± 2289	6835 ± 2889
	MG	1	3958	5859	6914
		2	5264	4320	5496
		3	3569	6139	2869
		Mean ± SD	4264 ± 888	5439 ± 979	5093 ± 2052
	CT	1	2872	7530	5941
		2	4357	3847	5681
		3	1666	8234	7181
		Mean ± SD	2965 ± 1348	6537 ± 2356	6268 ± 802
	UU	1	2097	4715	7148
		2	3534	4551	5925
		3	2170	4112	5838
		Mean ± SD	2600 ± 809	4459 ± 312	6304 ± 733
	UP	1	7725	6848	13455
		2	7948	8143	4505
		3	4230	11305	11991
		Mean ± SD	6634 ± 2085	8765 ± 2293	9984 ± 4801
	NG	1	2095	2790	2110
		2	2567	4117	2999
		3	2143	7031	6182
		Mean ± SD	2268 ± 260	4646 ± 2169	3764 ± 2141
	HSV-2	1	2259	1566	1824
		2	2219	1699	2326
		3	1086	3489	3113
		Mean ± SD	1855 ± 666	2251 ± 1074	2421 ± 650
Plasmid Mix	MH	1	1840	2137	1073
		2	1355	3426	2650
		3	1396	1424	2324
		Mean ± SD	1530 ± 269	2329 ± 1015	2016 ± 832
	MG	1	3867	4560	3577
		2	4481	4990	5032
		3	3357	4492	4161
		Mean ± SD	3902 ± 563	4681 ± 270	4257 ± 732
	CT	1	1067	3904	1492

	2	1075	3753	4779
	3	1083	1511	3112
	Mean ± SD	1075 ± 8	3056 ± 1340	3128 ± 1644
UU	1	2631	4190	938
	2	2357	3984	1572
	3	1517	3290	2405
	Mean ± SD	2168 ± 580	3821 ± 472	1638 ± 736
UP	1	1619	718	2268
	2	1191	3248	2916
	3	1167	2361	799
	Mean ± SD	1326 ± 254	2109 ± 1284	1994 ± 1085
NG	1	3369	2694	2758
	2	1021	660	3972
	3	2401	1630	2798
	Mean ± SD	2264 ± 1180	1661 ± 1017	3176 ± 690
HSV-2	1	5243	4587	1397
	2	1975	3867	5041
	3	3529	4361	3105
	Mean ± SD	3582 ± 1635	4272 ± 368	3181 ± 1823
IC	1	2766	798	943
	2	1760	N/A	5214
	3	2470	1776	1966
	Mean ± SD	2332 ± 517	1287 ± 692	2708 ± 2230

Table 7 Results of repeatability testing

Target	Level	Item	Size	Peak area	Lg Peak area
CT	H	mean	203.12	151214.90	5.18
		CV	0.02%	23.20%	1.72%
	L	mean	203.14	70411.35	4.83
		CV	0.02%	25.49%	2.77%
HSV-2	H	mean	273.88	261227.15	5.41
		CV	0.02%	16.00%	1.21%
	L	mean	273.90	167396.05	5.21
		CV	0.02%	26.34%	2.33%
MG	H	mean	185.92	266371.95	5.42
		CV	0.02%	10.12%	0.78%
	L	mean	185.95	156242.85	5.18
		CV	0.02%	22.91%	1.78%
MH	H	mean	172.86	125327.90	5.09
		CV	0.03%	23.53%	1.73%
	L	mean	172.86	66190.65	4.81
		CV	0.03%	26.35%	2.51%
NG	H	mean	252.04	137476.00	5.12
		CV	0.01%	31.34%	2.39%
	L	mean	252.03	76256.00	4.86
		CV	0.01%	31.37%	2.67%
UP	H	mean	233.80	110706.90	5.03
		CV	0.01%	25.16%	1.91%
	L	mean	233.78	57316.30	4.73
		CV	0.02%	35.34%	3.50%
UU	H	mean	216.33	69050.20	4.82
		CV	0.01%	29.56%	2.42%
	L	mean	216.32	42273.70	4.58
		CV	0.02%	34.52%	5.56%

Table 8 Resuls of specificity test

Cross pathogen	No.	Result
Staphylococcus aureus	JC1-1	negative（-）
	JC1-2	negative（-）
	JC1-3	negative（-）
Staphylococcus epidermidis	JC2-1	negative（-）
	JC2-2	negative（-）
	JC2-3	negative（-）
Pseudomonas aeruginosa	JC3-1	negative（-）
	JC3-2	negative（-）
	JC3-3	negative（-）
Klebsiella pneumoniae	JC4-1	negative（-）
	JC4-2	negative（-）
	JC4-3	negative（-）
Escherichia coli	JC5-1	negative（-）
	JC5-2	negative（-）
	JC5-3	negative（-）
Candida albicans	JC6-1	negative（-）
	JC6-2	negative（-）
	JC6-3	negative（-）
Human papillomavirus type 16	JC7-1	negative（-）
	JC7-2	negative（-）
	JC7-3	negative（-）
Human papillomavirus type 18	JC8-1	negative（-）
	JC8-2	negative（-）
	JC8-3	negative（-）

Table 9 Results of interference testing

Interferents	Final concentration	Number	Result
120mg/mLhemoglobin	1.2 mg/mL	GR1-1	CT+/UU+/UP+/MG+/MH+/NG+/HSV-2+
		GR1-2	CT+/UU+/UP+/MG+/MH+/NG+/HSV-2+
		GR1-3	CT+/UU+/UP+/MG+/MH+/NG+/HSV-2+
120mg/mL mucin	1.2 mg/mL	GR2-1	CT+/UU+/UP+/MG+/MH+/NG+/HSV-2+
		GR2-2	CT+/UU+/UP+/MG+/MH+/NG+/HSV-2+
		GR2-3	CT+/UU+/UP+/MG+/MH+/NG+/HSV-2+
1% clotrimazole	0.01 mg/mL	GR3-1	CT+/UU+/UP+/MG+/MH+/NG+/HSV-2+
		GR3-2	CT+/UU+/UP+/MG+/MH+/NG+/HSV-2+
		GR3-3	CT+/UU+/UP+/MG+/MH+/NG+/HSV-2+
2% miconazole	0.02mg/mL	GR4-1	CT+/UU+/UP+/MG+/MH+/NG+/HSV-2+
		GR4-2	CT+/UU+/UP+/MG+/MH+/NG+/HSV-2+
		GR4-3	CT+/UU+/UP+/MG+/MH+/NG+/HSV-2+
10% glycerol	0.1mg/mL	GR5-1	CT+/UU+/UP+/MG+/MH+/NG+/HSV-2+
		GR5-2	CT+/UU+/UP+/MG+/MH+/NG+/HSV-2+
		GR5-3	CT+/UU+/UP+/MG+/MH+/NG+/HSV-2+

Table 10 Statistical analysis of methodological consistency for CT

MPCE	Wuhan Easy Diagnosis		
	CT positive	CT negative	Total
CT positive	47	3	50
CT negative	8	147	155
Total	55	150	205

Table 11 Statistical analysis of methodological consistency for NG

MPCE	Wuhan Easy Diagnosis		
	NG positive	NG negative	Total
NG positive	49	7	56
NG negative	0	149	149
Total	49	156	205

Table12 Statistical analysis of methodological consistency for MG

MPCE	Wuhan Easy Diagnosis		
	MG positive	MG negative	Total
MG positive	50	9	59
MG negative	1	145	146
Total	51	154	205

Table13 Statistical analysis of methodological consistency for UU

MPCE	Wuhan Easy Diagnosis		
	UU positive	UU negative	Total
UU positive	23	11	34
UU negative	6	165	171
Total	29	176	205

Table14 Statistical analysis of methodological consistency for HSV-2

MPCE	Sansure Biotech		
	HSV-2 positive	HSV-2 negative	Total
HSV-2 positive	3	0	3
HSV-2 negative	0	202	202
Total	3	202	205

Table15 Statistical analysis of methodological consistency for MH

MPCE	Wuhan Easy Diagnosis		
	MH positive	MH negative	Total
MH positive	15	5	20
MH negative	1	184	185
Total	16	189	205

Table16 Statistical analysis of methodological consistency for UP

MPCE	Wuhan Easy Diagnosis		
	UP positive	UP negative	Total
UP positive	48	5	53
UP negative	4	148	152
Total	52	153	205

Table17 Capillary electrophoresis parameters overview

Parameter	Value
Oven temperature	60 ℃
Gel injection volume	120 uL
Current stabilization	5.0 ℃
Prerun voltage	15.0 kV
Prerun time	180 s
Injection voltage	3.5 kV
Injection time	8 s
Data delay time	240 s
Run voltage	19.5 kV
Run time	1120 s
Voltage ramp Steps	20 steps
Voltage ramp interval	15 s
