# Supplementary material for: Performance evaluation and application of a multiplex PCR capillary electrophoresis method for detecting nucleic acids of seven sexually transmitted pathogens
Source: Front Cell Infect Microbiol. 2026 May 18;16:1816857. doi: 10.3389/fcimb.2026.1816857 (PMC13223153; doi:10.3389/fcimb.2026.1816857)
Supplement: Supplementary file 2 [file Table2.pdf]

1、Single PCR capillary electrophoresis profile

Figure 1 The results of negative sample

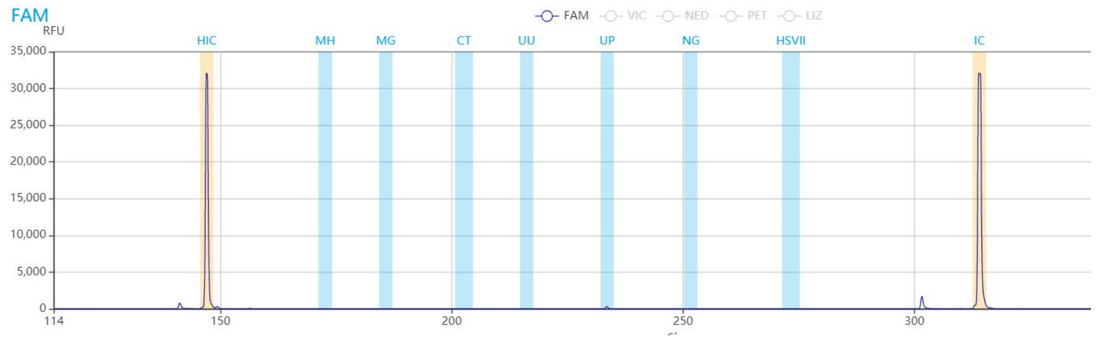

Figure 2 The results of the negative control

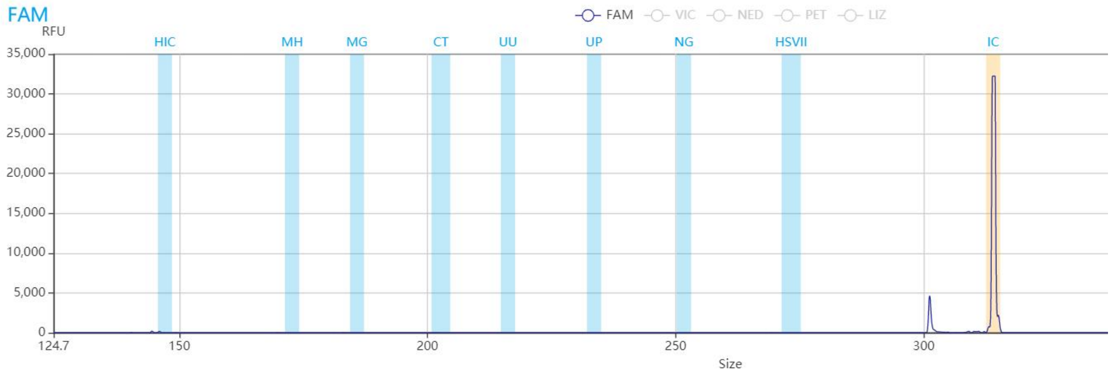

Figure 3. Positive characteristic peaks of MH and IC

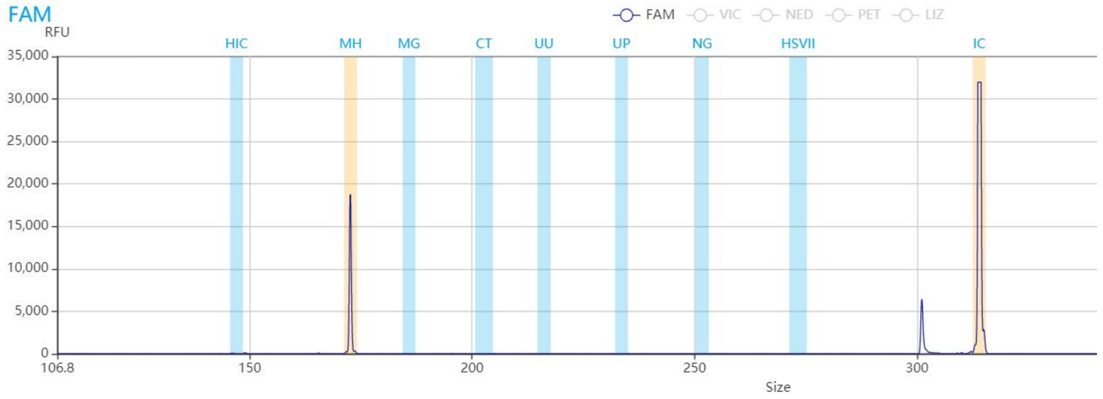

Figure 4 Positive characteristic peaks of MG and IC

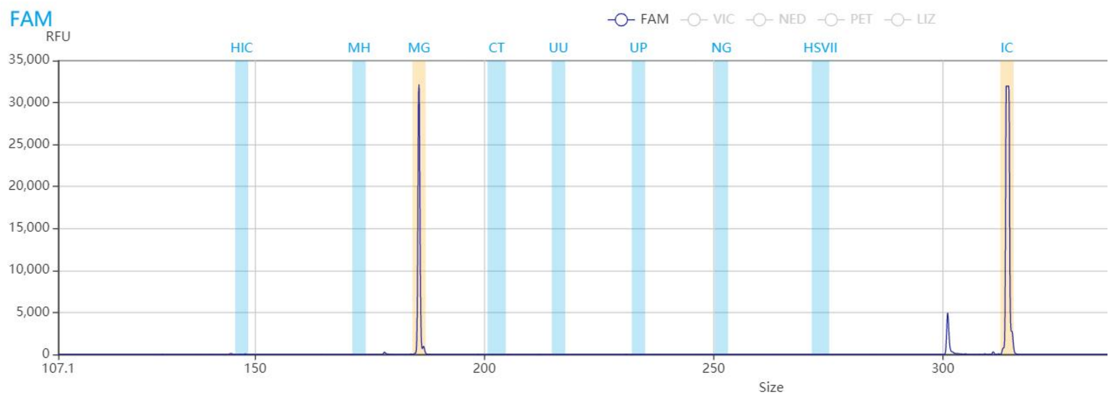

Figure 5 Positive characteristic peaks of CT and IC

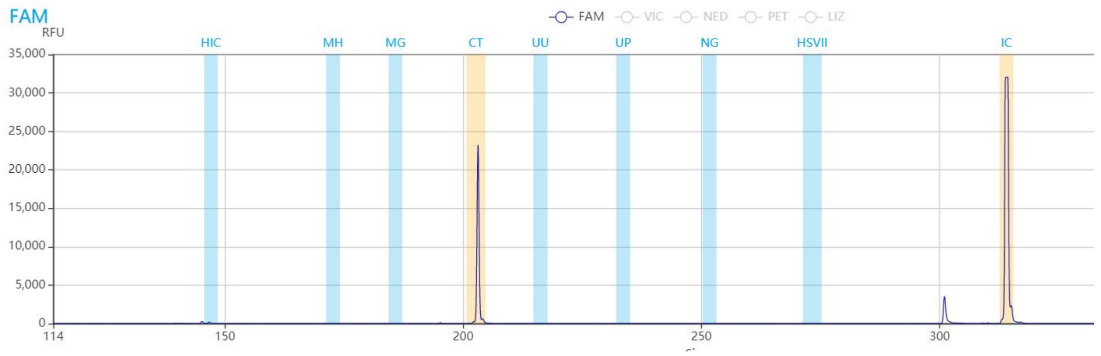

Figure 6. Positive characteristic peaks of UU and IC

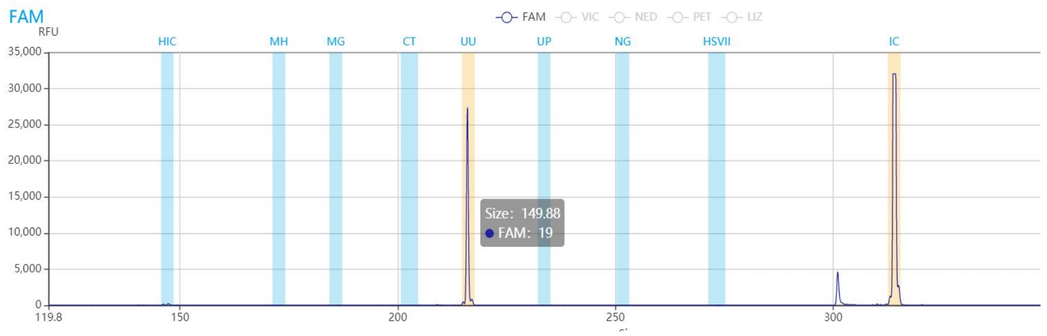

Figure 7 shows positive characteristic peaks of UP and IC

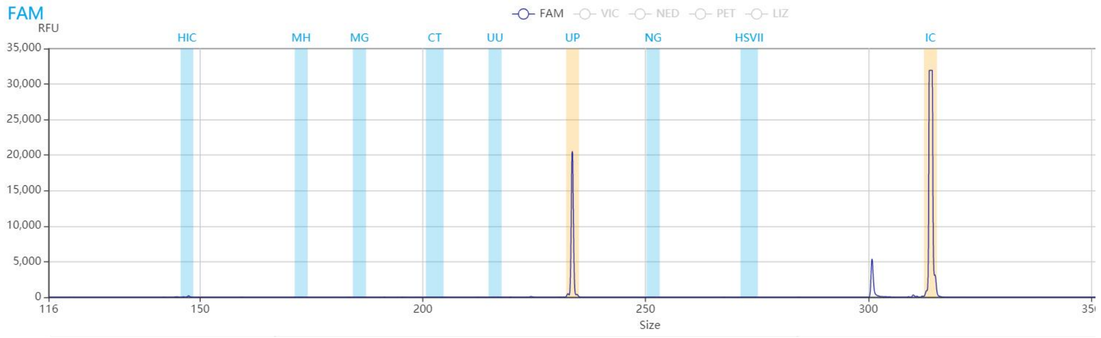

Figure 8. Positive characteristic peaks of NG and IC

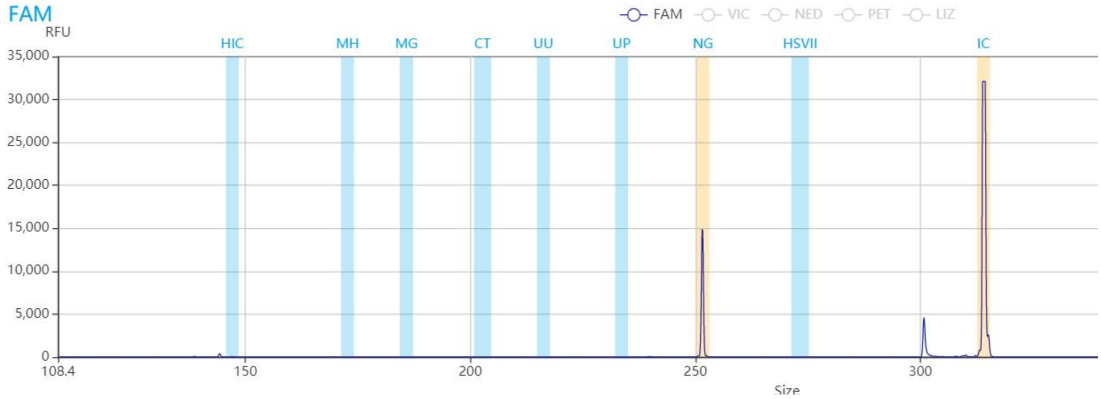

Figure 9. Positive characteristic peaks of HSVII and IC

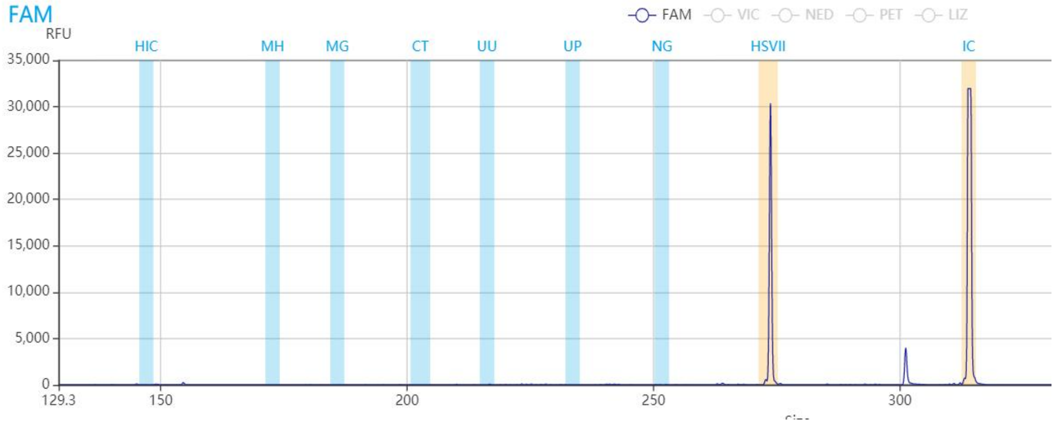

2、Agarose gel electrophoresis pattern of PCR for recombinant colonies of simulated pathogen plasmids.

Figure 10. Gel electrophoresis pattern of PCR products from recombinant colonies of CT plasmid

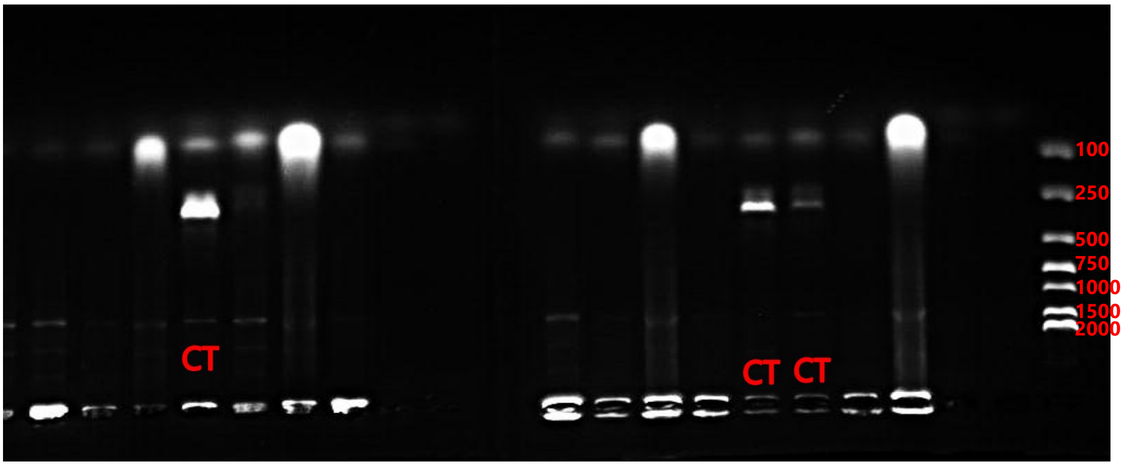

Figure 11. PCR electrophoresis pattern of recombinant colonies of UP plasmid

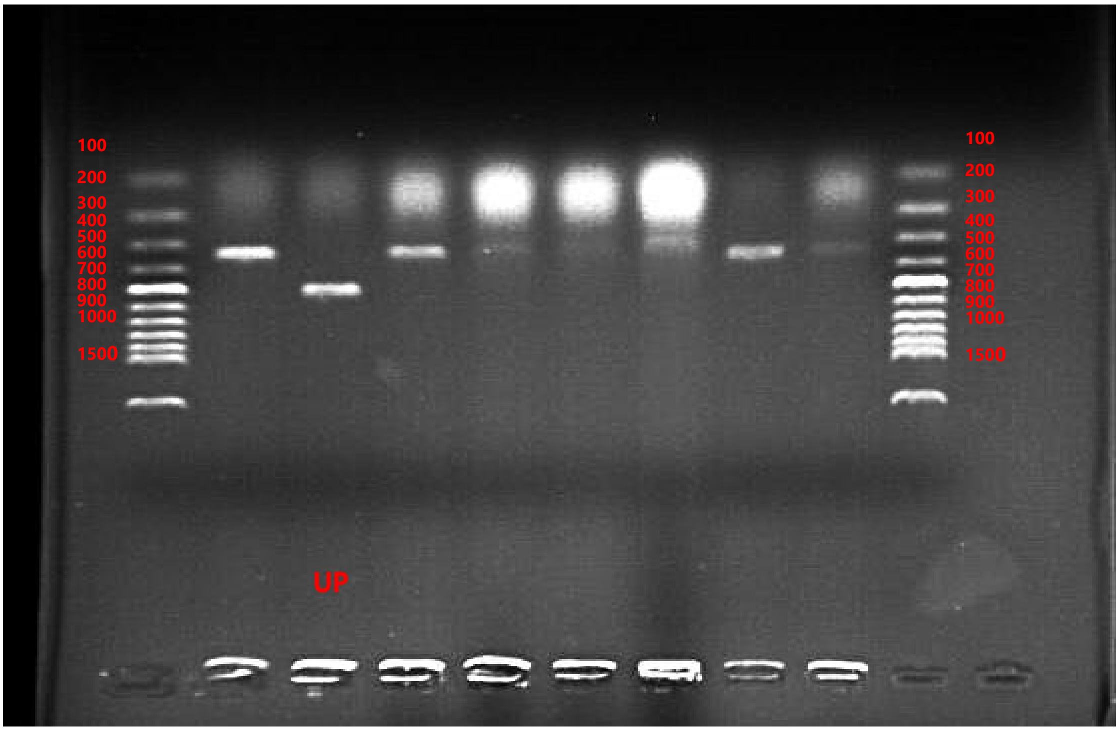

Figure 12. PCR electrophoresis pattern of recombinant colonies of HSV-2 plasmid

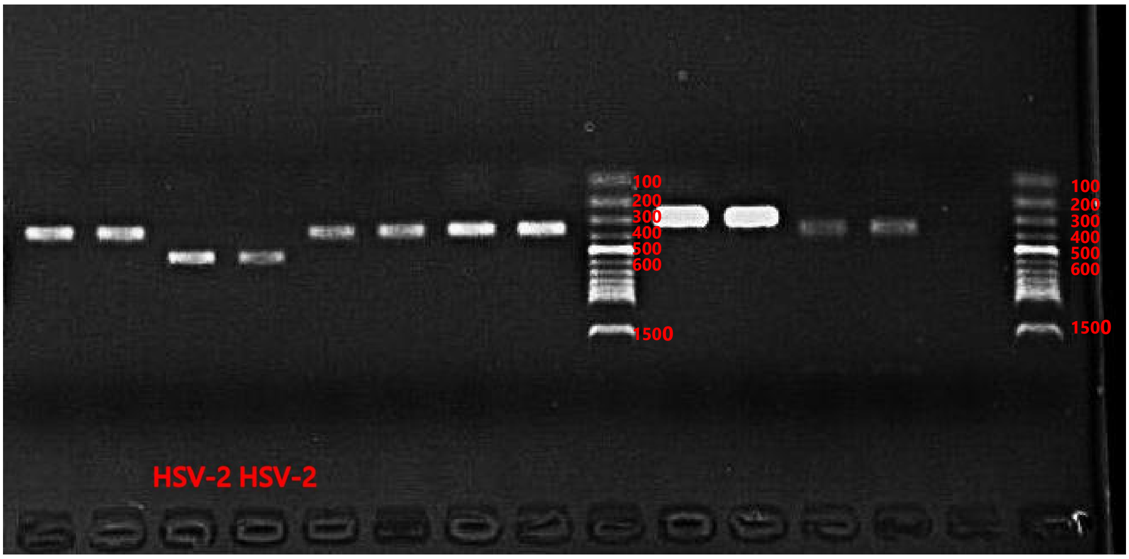

Figure 13. PCR electrophoresis pattern of recombinant colonies of UU plasmid

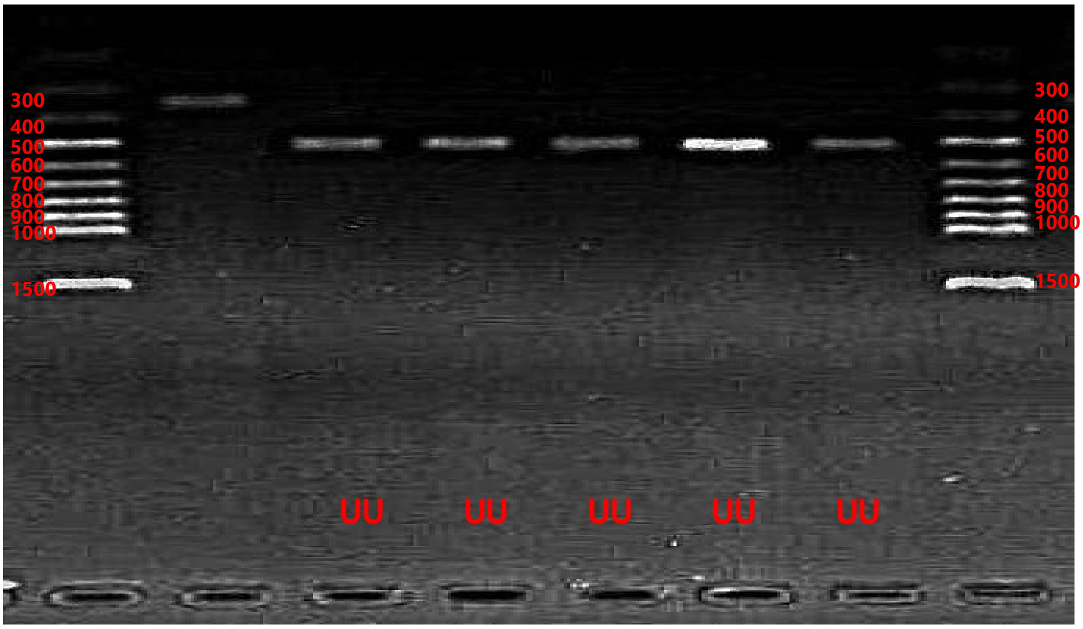

Figure 14. PCR electrophoresis pattern of recombinant colonies of MH plasmid

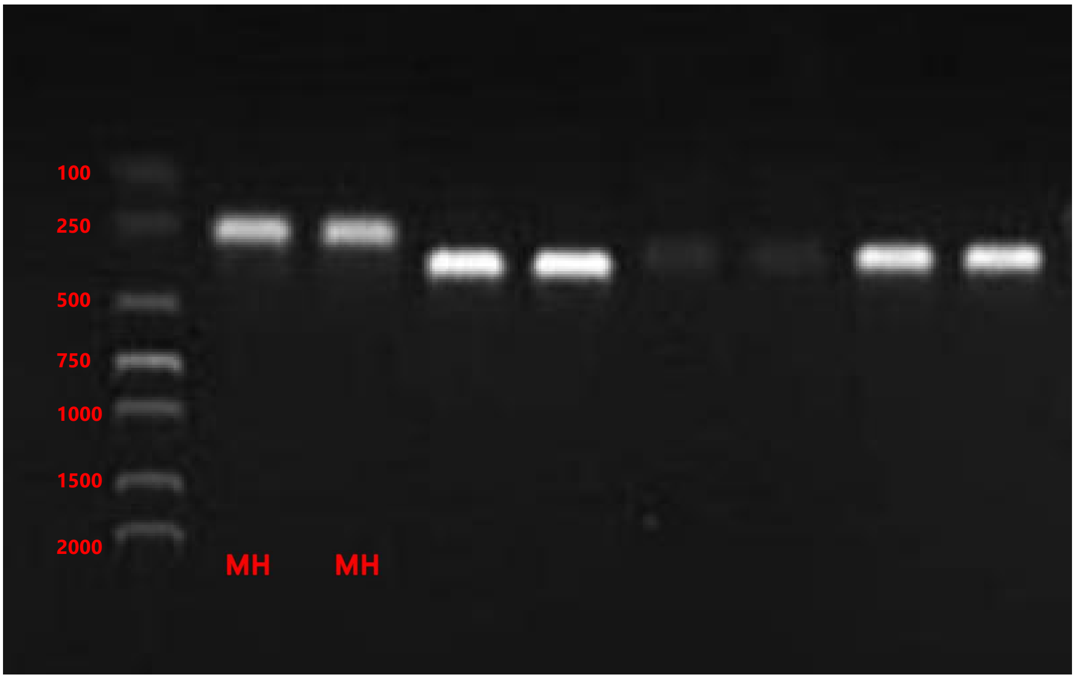

Figure 15. PCR electrophoresis pattern of recombinant colonies of NG plasmid

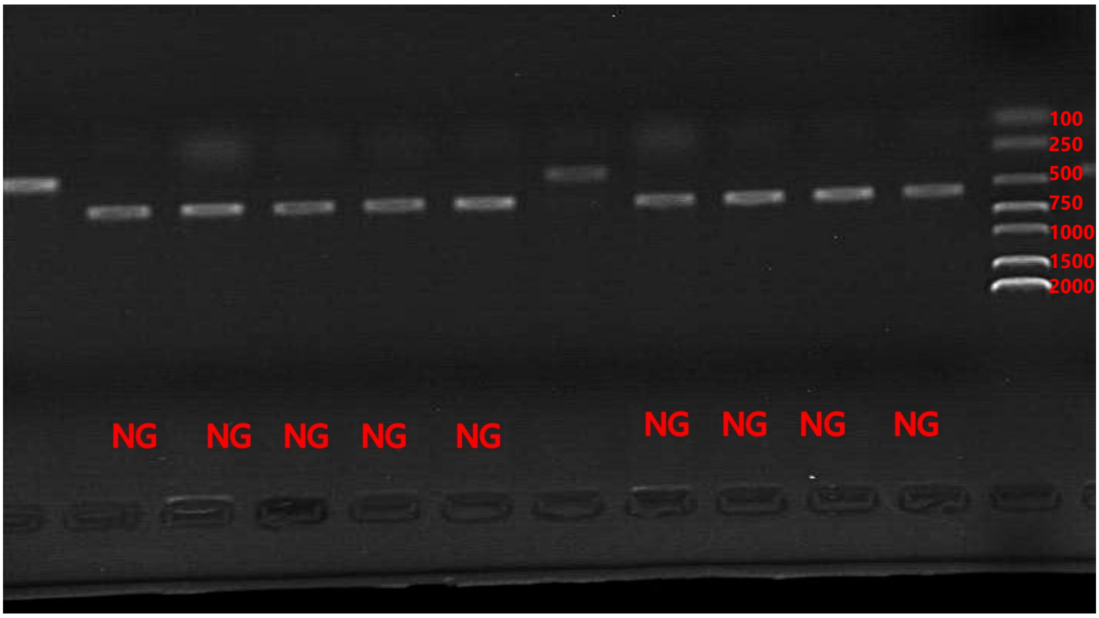

Figure 16. PCR electrophoresis pattern of recombinant colonies of MG plasmid

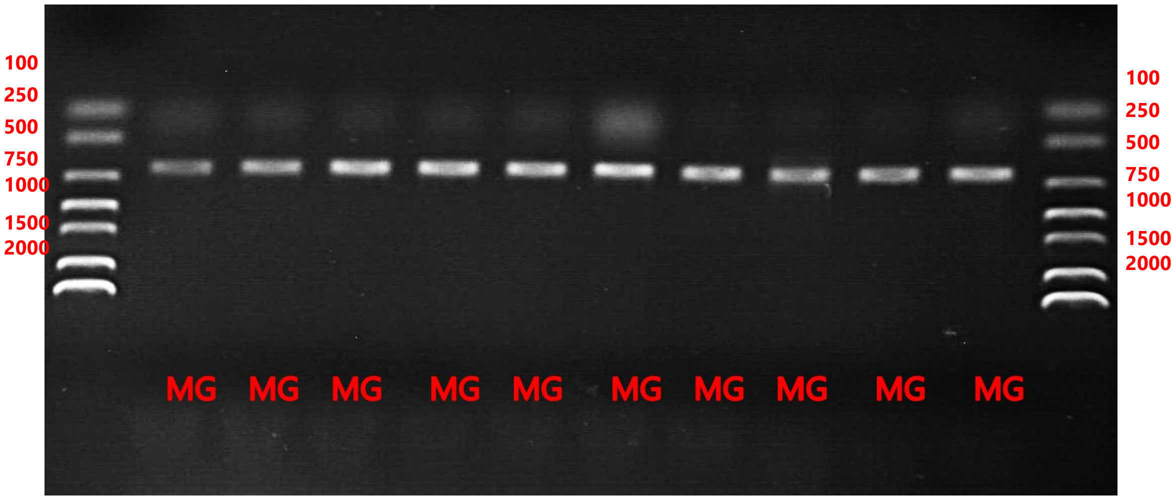

3、Sequencing results of the simulated pathogen recombinant plasmid

Figure 17 Sequencing results of CT recombinant plasmids

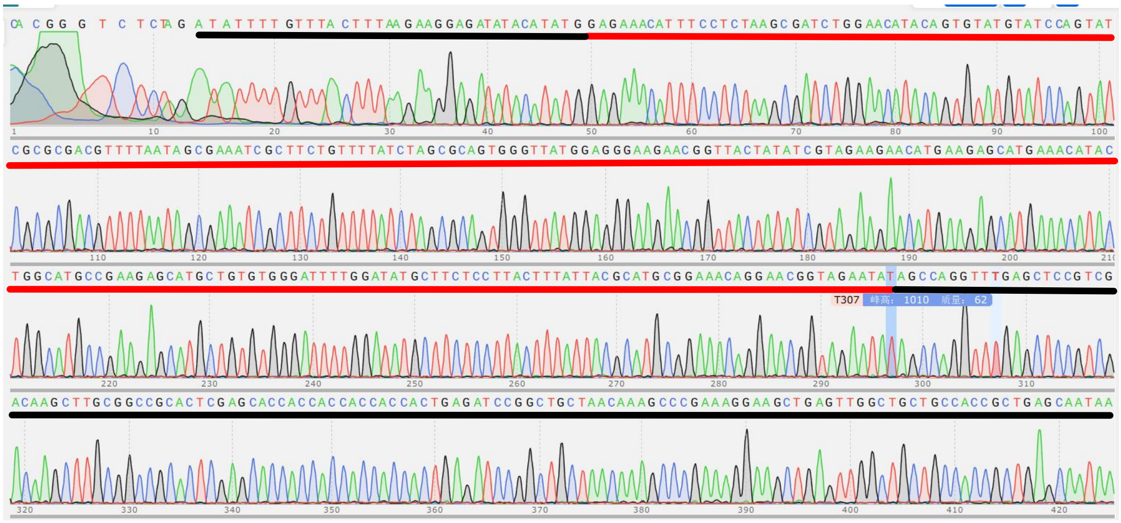

In Figure 17, the region marked by the red line (50-307 bp) represents the CT target sequence, while the region marked by the black line represents the vector sequence.

Figure 18 Sequencing results of HSV-2 recombinant plasmids

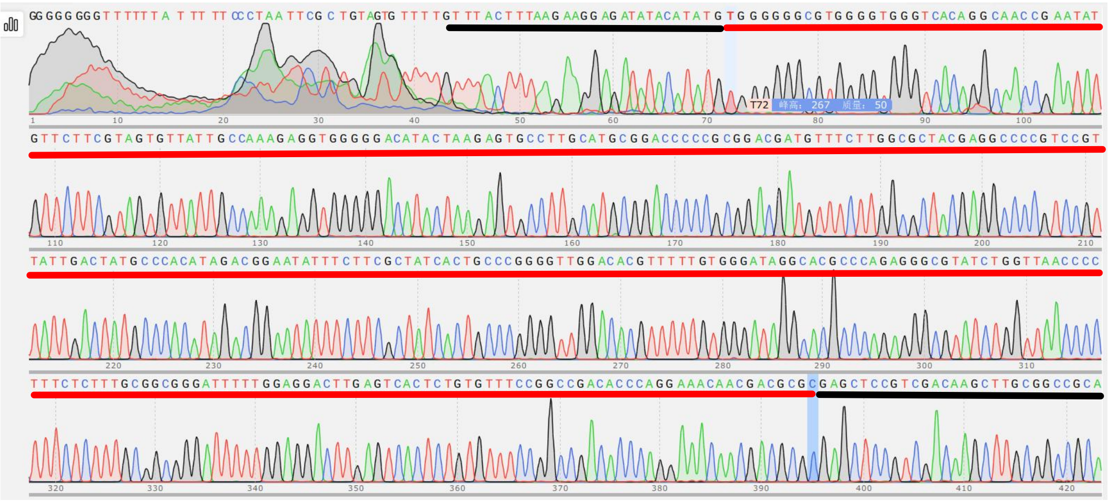

In Figure 18, the red line area (72-395 bp) represents the HSV-2 target sequence, and the black line area represents the vector sequence.

Figure 19 Sequencing results of UU recombinant plasmids

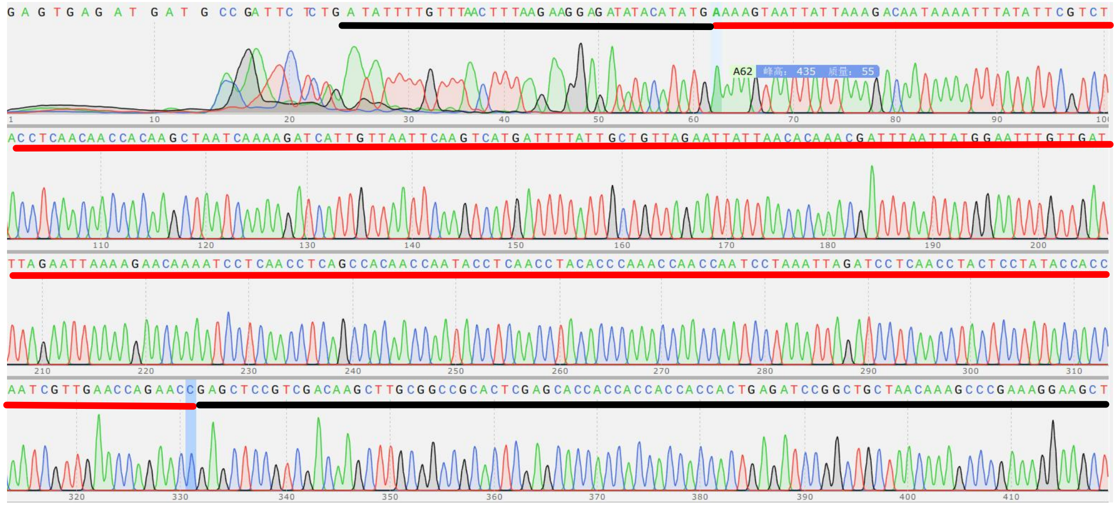

In Figure 19, the region marked by the red line (62-331 bp) represents the UU target sequence, while the region marked by the black line represents the vector sequence.

Figure 20 Sequencing results of UP recombinant plasmids

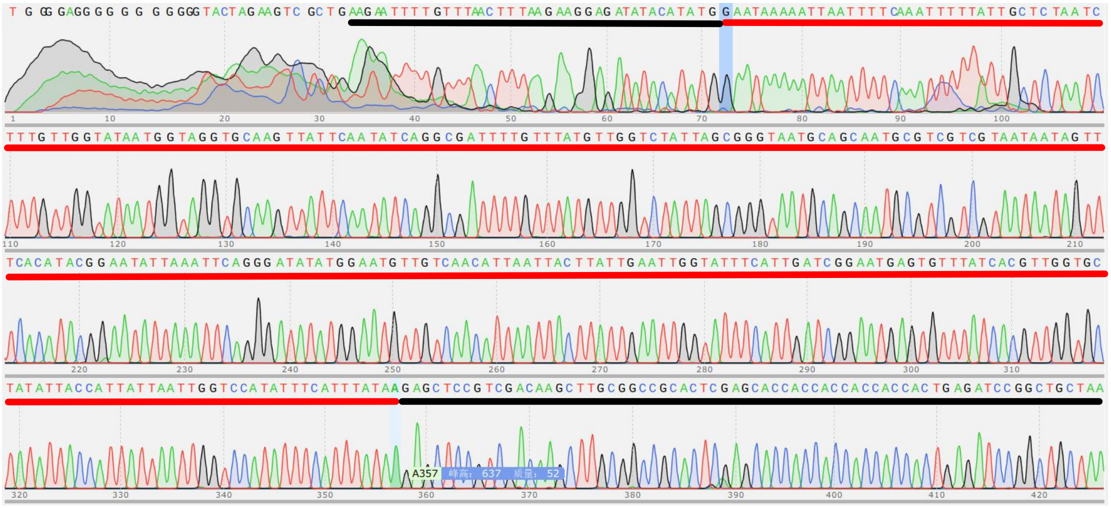

In Figure 20, the region marked by the red line (72-357 bp) represents the UP target sequence, while the remaining region marked by the black line represents the vector sequence.

Figure 21 Sequencing results of NG recombinant plasmids

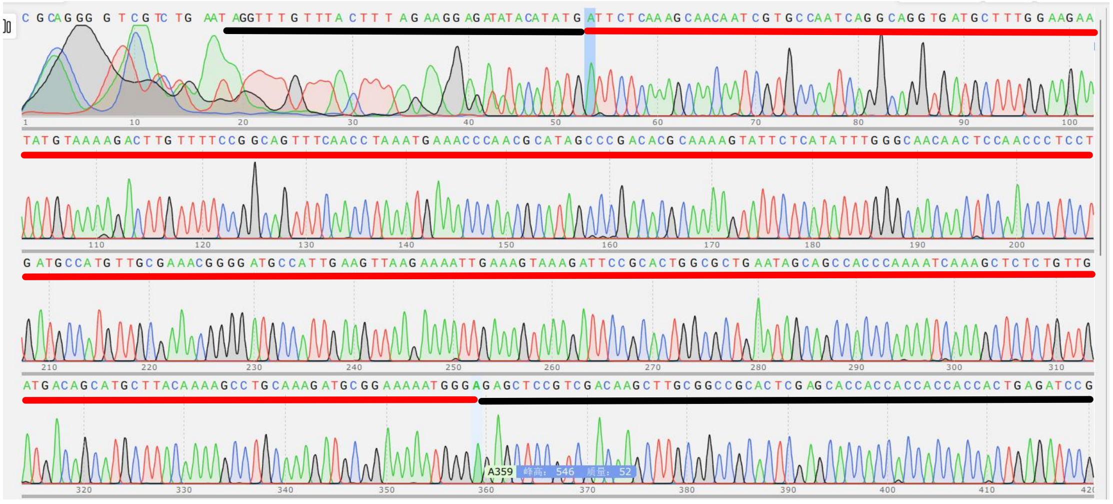

In Figure 21, the red line area (53-359 bp) represents the NG target sequence, and the remaining black line area represents the vector sequence.

Figure 22 Sequencing results of MG recombinant plasmids

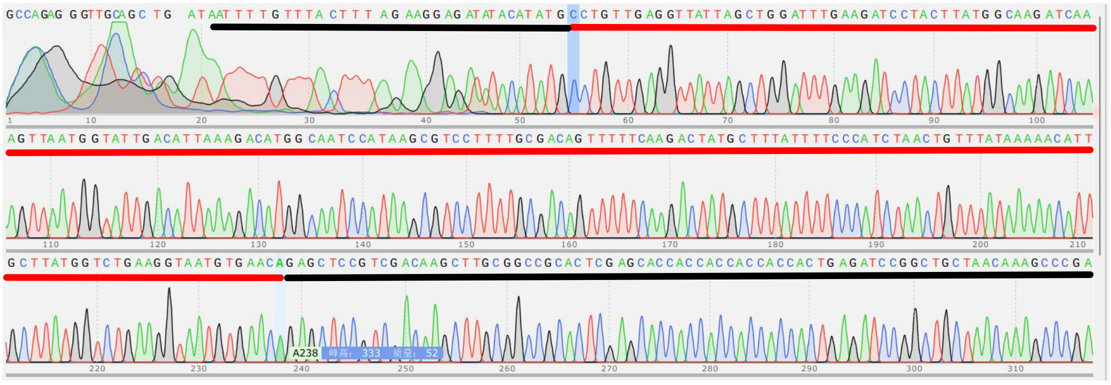

In Figure 22, the red line area (55-238 bp) represents the MG target sequence, while the remaining black line area represents the vector sequence.

Figure 23 Figure 22 Sequencing results of MH recombinant plasmids

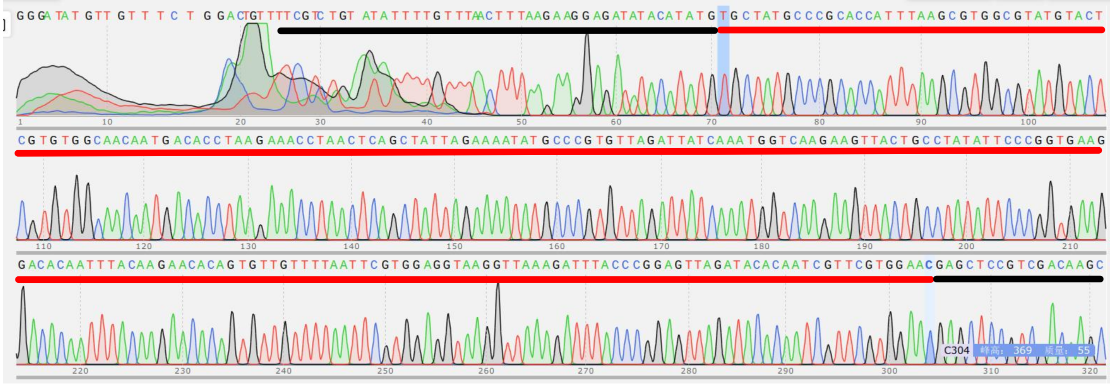

In Figure 23, the region marked by the red line (71-304 bp) represents the MH target sequence, while the remaining region marked by the black line is the vector sequence.
